# Mental health of non-binary youth: a systematic review and meta-analysis

**DOI:** 10.1186/s13034-024-00822-z

**Published:** 2024-10-09

**Authors:** Diana Klinger, Sofia-Marie Oehlke, Stefan Riedl, Ken Eschbaum, Heidi Elisabeth Zesch, Andreas Karwautz, Paul L. Plener, Oswald D. Kothgassner

**Affiliations:** 1 Department of Child and Adolescent Psychiatry, Medical University of Vienna, Vienna, Austria; 2 Comprehensive Center for Pediatrics, Medical University of Vienna, Vienna, Austria; 3 Department of Pediatrics and Adolescent Medicine, Medical University of Vienna, Vienna, Austria; 4 St. Anna Children’s Hospital, Medical University of Vienna, Vienna, Austria; 5 Department of Child and Adolescent Psychiatry and Psychotherapy, University of Ulm, Ulm, Germany

**Keywords:** Non-binary, Mental health, Adolescence, Youth, Transgender, Cisgender, Meta-analysis

## Abstract

**Background:**

Non-binary identities are increasingly recognized within the spectrum of gender diversity, yet there is a dearth of research exploring the mental health challenges specific to this population. Therefore, this systematic review and meta-analysis aimed to comprehensively assess the mental health outcomes of non-binary youth in comparison to their transgender and cisgender peers.

**Methods:**

A systematic search was conducted to identify relevant studies across three electronic databases (PubMed, Scopus, Web of Science) covering the period from inception to October 2023. The meta-analysis was performed employing a random-effects model. Inclusion criteria encompassed studies comparing non-binary youth with transgender or cisgender youth, providing data on mental health outcomes such as general mental health, depressive and anxiety symptoms, self-harm and suicidality.

**Results:**

Twenty-one studies, meeting the inclusion criteria and originating from six different countries, were included in the analysis. The sample encompassed 16,114 non-binary, 11,925 transgender, and 283,278 cisgender youth, with ages ranging from 11 to 25 years. Our meta-analysis revealed that non-binary youth exhibit significantly poorer general mental health compared to both transgender (*d* = 0.24, 95% CI, 0.05–0.43, *p* =.013) and cisgender youth (*d* = 0.48, 95% CI, 0.35–0.61, *p* <.001), indicating a more impaired general mental health in non-binary youth. Regarding depressive symptoms, when comparing non-binary and cisgender individuals, a moderate and significant effect was observed (*d* = 0.52, 95% CI, 0.41–0.63, *p* <.001). For anxiety symptoms, a small but significant effect was observed in the comparison with cisgender individuals (*d* = 0.44, 95% CI, 0.19–0.68, *p* =.001). Furthermore, non-binary individuals exhibited lower rates of past-year suicidal ideation than transgender peers (OR = 0.79, 95% CI, 0.65–0.97, *p* =.023) and higher rates of lifetime suicidal ideation than cisgender youth (OR = 2.14, 95% CI, 1.46–3.13, *p* <.001).

**Conclusion:**

Non-binary youth face distinct mental health challenges, with poorer general mental health, elevated depressive and anxiety symptoms compared to cisgender, and similar rates of self-harm and suicidal behavior compared to transgender individuals. These findings underscore the urgent need for targeted interventions, including gender-affirming mental health support, to address the specific needs of non-binary youth.

**Supplementary Information:**

The online version contains supplementary material available at 10.1186/s13034-024-00822-z.

## Background

The conventional binary conceptualization of gender, which categorizes individuals into female and male categories, is progressively giving way to a more nuanced understanding. Gender is increasingly recognized as a spectrum, encompassing a growing number of individuals, particularly among adolescents and young adults, who identify themselves within a broader gender spectrum [1]. Gender identity refers to the individual’s inner sense of belonging to a specific gender [2]. Those who identify as non-binary experience their gender identity beyond the binary gender framework, with the term “non-binary” encompassing a spectrum of diverse identity experiences. Individuals with a non-binary gender identity may adopt multiple gender identities at the same or different times (e.g., “bigender”), have no specific gender identity or hold a neutral one (e.g., “agender”), have a gender identity that includes or combines elements from different genders (e.g., “genderqueer”, “polygender”), or undergo changes in their gender identity over time (e.g., “genderfluid”). It’s important to note that the list provided is not exhaustive, as individual experiences of gender identity can vary across different contexts [1, 3].

A non-binary gender identity, like all other gender identities (including cisgender, transgender etc.), is a normal variant of gender identity and is not inherently in need of treatment. In this review and meta-analysis, the term “cisgender” refers to individuals, whose gender identity aligns with the sex they were assigned at birth (e.g., male or female), identifying with the gender traditionally associated with their biological or anatomical sex. In contrast, the term “transgender” includes individuals whose gender identity differs from their assigned sex at birth, often identifying themselves with the opposite binary gender, though not exclusively [3, 4].

While some non-binary individuals may not have specific preferences concerning their social interactions or physical sex characteristics, there are many individuals who cannot identify with names or pronouns that carry female or male connotations and prefer to be addressed with different names or pronouns. Additionally, a subgroup of non-binary individuals may experience a discrepancy between their gender identity and physical characteristics, which is referred to as gender incongruence (GI). When this incongruence results in significant distress, it is termed as gender dysphoria (GD). In such cases, non-binary individuals may meet the diagnostic criteria for GI or GD according to the International Classification of Diseases, 11th Revision (ICD-11; [5]) and/or the Diagnostic and Statistical Manual of Mental Disorders, 5th Edition (DSM-V; [6]), respectively. GI/GD can, in non-binary individuals, similar to transgender individuals, be associated with a need for gender-affirming treatment. The specific gender-affirming interventions requested vary from person to person. Some non-binary individuals may perceive a particular form of treatment, such as gender-affirming hormone treatment or chest masculinizing surgery, as necessary, while others may not [1, 7].

While comprehensive data on the prevalence of non-binary identity remain limited, recent studies indicate a growing recognition and representation of non-binary individuals, particularly among adolescents and young adults. The prevalence of non-binary identity can significantly vary depending on the studied population and the methodology employed to assess gender. Most research on non-binary populations has focused on adults, and studies have revealed a range of 1.2–4.6% self-identifying as non-binary in the general population [8–10], with higher rates ranging from 18.5 to 50% in gender-diverse populations [11, 12], and approximately 8.2–14.3% among individuals seeking gender specific medical treatment [13, 14]. Among youth up to 25 years old, prevalence ranges from 2.9 to 9% in general population surveys [15, 16] increasing to 41–53.4% in gender-diverse youth populations [17, 18]. However, among youth seeking care at gender clinics, about 11–25.6% identify as non-binary [19–21]. These findings underscore the importance of recognizing and understanding the diverse landscape of non-binary identity across various age groups and populations.

To date, there has been limited research specifically focused on non-binary youth, especially regarding their mental health. Most studies have concentrated on transgender and/or gender dysphoric children and adolescents, often including non-binary individuals within the broader transgender, gender diverse or gender dysphoric group [22–24]. A distinction in data analysis would be crucial, given the evidence of more adverse mental health outcomes among transgender and gender-diverse youth in general [25], alongside the well-documented additional societal challenges faced by non-binary individuals, including heightened barriers to accessing healthcare services [1, 3, 7]. In their study, de Graaf et al. [26] examined the psychological well-being of non-binary individuals across different age groups. In the adolescent cohort, a stronger association between non-binary gender identity and psychological problems emerged, particularly for those assigned female at birth. In the adult sample, experiencing psychological difficulties was related to a more pronounced non-binary identity and a younger age. The first and so far, only systematic review conducted by Chew et al. [27] investigated the sociodemographic and clinical characteristics of non-binary youth, making comparisons to their cisgender and transgender counterparts. Despite limited data, primarily from five studies addressing the psychological profile of non-binary youth, the review revealed several key findings: non-binary youth exhibited concerning mental health outcomes, including elevated rates of depression, anxiety, and suicidal thoughts, which often paralleled or exceeded those observed in transgender and binary youth. They encountered reduced support and a higher risk of abuse and victimization compared to their cisgender peers. Additionally, when compared with transgender and binary youth, non-binary individuals faced greater challenges in accessing specialized healthcare services.

Considering the scarcity of research in this field, the aim of this review and meta-analysis is to evaluate and compare the mental health outcomes of non-binary youth in relation to their cisgender and binary transgender counterparts. Through a systematic examination of the general mental health and selected mental health indicators, such as depressive and anxiety symptoms, self-harm and suicidality in non-binary youth, the primary objective of this study is to contribute valuable insights into the unique challenges faced by non-binary adolescents and young adults, providing a basis for targeted interventions and support tailored to their specific needs within the broader context of youth mental health.

## Methods

This systematic review and meta-analysis is reported in accordance with the Preferred Reporting Items for Systematic Reviews and Meta-Analyses (PRISMA) guidelines (Additional file 1: Table S1) [28].

### Data sources and search strategy

A systematic search was conducted for published articles across three electronic databases: PubMed, Scopus, and Web of Science, covering the period from their inception to October 27, 2023.

The search strategy encompassed key search terms for the population (non-binary children, adolescents, and young adults aged ≤ 25 years) and for mental health as outcome. Key search terms employed included variations of “non-binary” (non*binary OR nonbinary), terms associated with age (child* OR adolesc* OR youth* OR young adult*), and mental health. Various iterations of search terms were trialed, including hyphenated or spaced versions (e.g., “non-binary” or “non binary”), as well as alternative terminology like “psychological problems” or “psychological functioning” regarding mental health. However, these modifications yielded negligible impacts on search outcomes and did not deliver further relevant results. The final strategy combined subject-specific terminology with everyday language, age filters and underwent multiple revisions for optimization. Initially developed in PubMed, it was subsequently applied across the other databases. To expand our search, we also reviewed previous systematic reviews and meta-analyses on the relevant topics and checked the reference lists of included studies, resulting in the identification of four additional references.

### Inclusion and exclusion criteria

The inclusion criteria for this study encompassed quantitative research studies featuring participants with gender identities categorized as non-binary, with separate data available for this group, and comparisons made with transgender or cisgender individuals. Furthermore, the studies were required to include data for children, adolescents or young adults within the age range of 0 to 25 years. Additionally, eligible studies were required to contain pertinent information concerning general mental health or selected mental health outcomes such as depression, anxiety, self-harm, and suicidality, aligning with the study’s comprehensive focus on mental health aspects.

Studies that did not report mental health measures for non-binary individuals separately or did not make comparisons with transgender or cisgender individuals were excluded from this review and meta-analysis. Additionally, studies were excluded if the age of the sample exceeded 25 years without including a distinct subgroup under 25 years for potential analysis. Qualitative studies, case studies, reviews, guidelines, conference notes were also not considered, as they did not report sufficient quantitative data at group level. Furthermore, studies reported in languages other than English were excluded as well.

### Screening and selection process

The screening and selection process was conducted independently by two reviewers (DK and S-MO). As the first step, duplicates were removed, followed by the screening of titles and abstracts of the remaining articles to identify those meeting the predetermined inclusion criteria. Subsequently, full texts of the potentially eligible articles were assessed for final inclusion. In cases of disagreements or uncertainty about eligibility, consensus was reached through discussion.

### Data extraction

Data extraction was carried out by two independent reviewers (DK and KE), who systematically collected the following information from the selected studies: study characteristics (including first author, publication year, country of origin, sample size, subgroups of participants and the total score resulting from the quality assessment), population characteristics (including demographics and treatment status related to gender-affirming treatment), and assessed mental health outcomes (encompassing measures of general mental health, depression, anxiety, self-harm, and suicidality).

### Quality assessment

Study quality and potential bias were assessed independently by two raters (S-MO and KE). In cases where the two raters could not reach a consensus, a third rater (DK) was consulted for resolution. The assessment of studies was conducted using a critical appraisal tool designed for Analytical Cross-Sectional Studies, developed by the Joanna Briggs Institute (JBI) [29]. This tool comprised eight questions, each with response options of “yes”, “no”, “unclear,” or “not applicable”. To evaluate the risk of bias and methodological quality, the number of positive responses to each question was totaled, with a score of one assigned for “yes” and zero for any other response category. Subsequently, scores were converted into percentages, with studies scoring ≤ 49%, 50–69%, and ≥ 70% classified as having high, moderate, or low risk of bias, respectively [30, 31]. The total summed scores and the corresponding percentages for each study can be found in Additional file 1: Table S2, providing an evaluation of study quality and potential bias in the included research.

### Data synthesis and statistical analysis

Meta-analyses were conducted to compare non-binary individuals with transgender and/or cisgender counterparts across various mental health domains, including general mental health, depression, anxiety, self-harm, and suicidality. In total, 13 separate meta-analyses were performed, with each focusing on a specific mental health outcome and comparison group (non-binary vs. transgender or non-binary vs. cisgender). Separate meta-analyses were necessary due to variations in group comparisons, with some studies including all three groups (cisgender, transgender, and non-binary) and others only two. Additionally, the heterogeneity in outcome measures across studies required distinct analyses for each mental health domain.

The analyses were performed using Comprehensive Meta-Analysis (CMA) software Version 4 [32], which was utilized for the entire analytical process, encompassing data entry, statistical analysis, and plot generation. For a meta-analysis to be carried out, a minimum of three studies on a particular topic, with separate data on the relevant populations (non-binary vs. transgender or non-binary vs. cisgender), was required. Some studies presented data partially or comprehensively separated based on the assigned sex at birth. When data were reported separately for both compared populations and pooled effect sizes did not significantly differ for each subgroup (assigned female at birth [AFAB] and assigned male at birth [AMAB]), the data were merged for analysis. When data were reported only partially separated, an Excel spreadsheet (provided by Biostat, Inc.) was used for initial merging, with subsequent entry into the CMA spreadsheet. If necessary, the program automatically converted the continuous or dichotomized data to compute the selected effect size.

To account for the variation in scales and methods used across studies reporting data on general mental health, depression, and anxiety, standardized mean difference (Cohen’s d) was calculated. Effect sizes of 0.2, 0.5, and 0.8 were considered indicative of small, moderate, and large effects, respectively [33]. In the analysis of general mental health among the compared populations, when multiple subscales of the same questionnaire were reported, they were integrated into a single outcome measure using CMA.

For investigation of differences in the prevalence of self-harm and suicidality among non-binary and transgender/cisgender youth, odds ratios (OR) and their corresponding 95% confidence intervals were used as statistical measures. Outcome measures combining rates on self-harm and suicidality were excluded from the meta-analysis. For self-harm, suicidal ideation, and suicide attempts, we conducted separate analyses for lifetime and past-year prevalence.

Due to the diverse study characteristics, we applied a random-effects model to account for variations among the included studies. Heterogeneity among the studies was assessed using forest plots and Cochran Q statistic *p* values. A *p*-value below the 0.05 threshold indicated substantial heterogeneity, while *I*^*2*^ values were calculated to quantify the proportion of heterogeneity, with the latter categorizing it as low (25%), moderate (50%), or high (75%) [34].

## Results

### Study selection

**Fig. 1:**
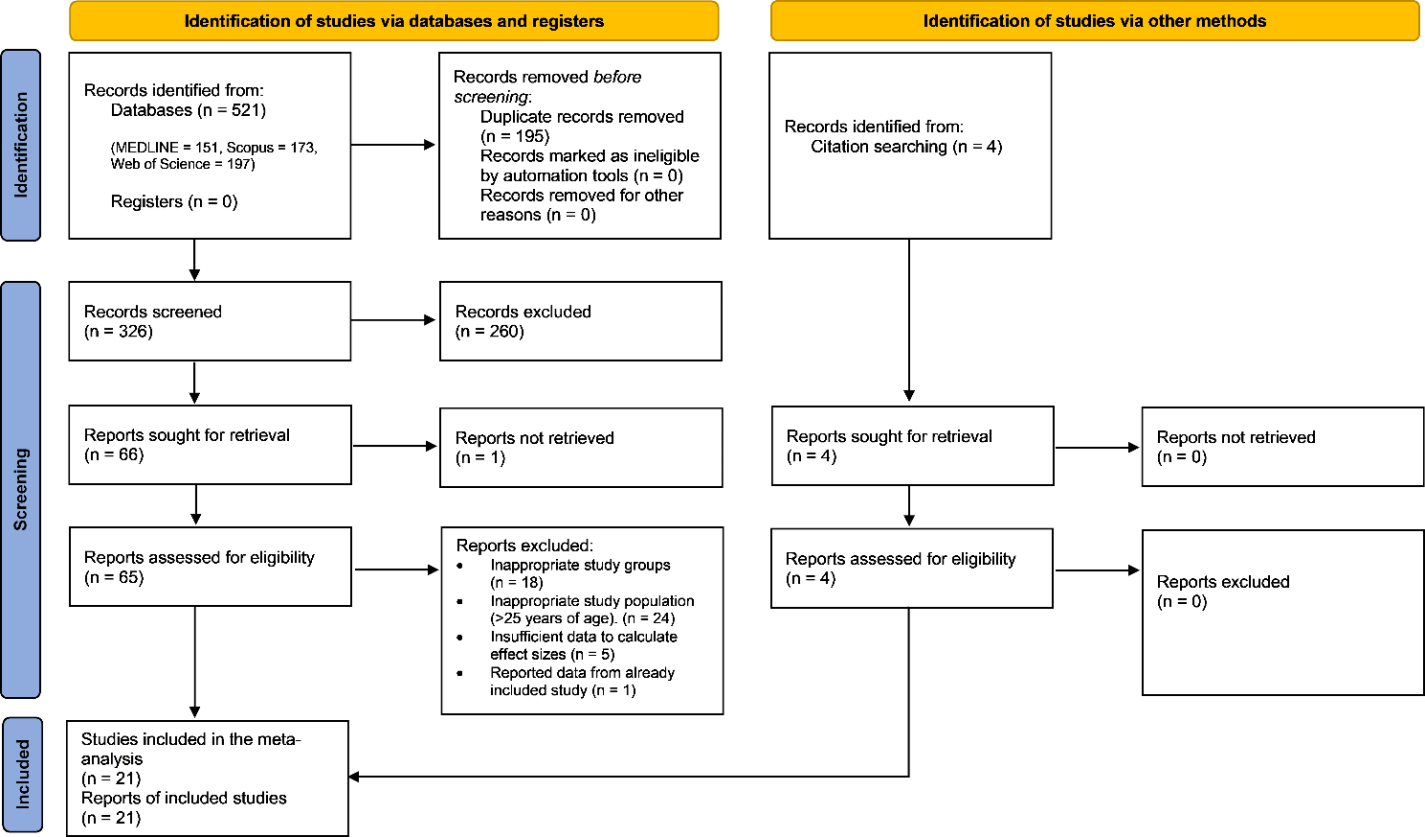
PRISMA flow diagram

A total of 521 records were initially identified from three electronic databases: Medline (*n* = 151 records), Scopus (*n* = 173 records), and Web of Science (*n* = 197 records). After removing 195 duplicate records, the titles and abstracts of the remaining 326 records were screened, leading to the exclusion of 260 records. Subsequently, full text of 65 reports were retrieved for further evaluation. Reasons for exclusion after full-text screening were recorded and are detailed in Additional file 1: Table S3. In addition, four articles were identified through a search in the reference lists of relevant publications. Ultimately, a total of twenty-one articles met the inclusion criteria and were included in the meta-analyses. The detailed screening process is outlined in the PRISMA flow diagram (Fig. 1).

### Study characteristics

The characteristics of the included studies are presented in Table 1. The total sample size across the studies was 322,602, comprising 16,114 non-binary individuals, 11,925 transgender individuals, and 283,278 cisgender individuals. The age range of the study participants was between 11 and 25 years. Only three out of the twenty-one studies provided information regarding treatment status related to gender-affirming treatment [17, 35, 36]. The publication years of the included studies ranged from 2017 to 2023. All of the studies included were either cross-sectional studies or longitudinal studies that reported cross-sectional data.

General mental health was assessed in six studies, each comparing non-binary and transgender individuals [16–18, 37–39], with three of them also including comparisons with cisgender individuals [16, 37, 38]. Aparicio-Garcia et al. [16] used the General Health Questionnaire (GHQ-12; [40]), Childs et al. [38] six clinical subscales of the Behavior Assessment System for Children, Third Edition, Self-Report of Personality-Adolescent (BASC-3 SRP-A; [41]), Ciria-Barreiro et al. [37] the HBSC Symptom Checklist’s Psychological Complaints subscale [42], Rusow et al. [39] three subscales of the Brief Symptom Inventory-18 (BSI-18; [43]), whereas Clark et al. [17] and Rimes et al. [18] surveys included a single item concerning general mental health, respectively.

Depressive symptoms were analyzed in a total of fourteen studies, with thirteen of them comparing non-binary and transgender youth [35, 36, 38, 39, 44–52]. Among these thirteen studies, seven also included cisgender youth for comparative analysis [38, 44, 45, 49–52]. One study reported a comparison between non-binary and cisgender youth [53]. In four studies [46, 48, 50, 51], depressive symptoms were operationalized using different versions of the Center for Epidemiologic Studies Depression Scale (CES-D; [54]), the CES-D-4, the CES-D-9 and the CES-D-10, respectively. Two studies utilized different versions of the Patient Health Questionnaire (PHQ) [52, 53]. Wang et al. [52] employed the PHQ-2 [55], while Kaltiala et al. [53] used the PHQ-9 [56]. Childs et al. [38] used the Depression subscale of the BASC-3 SPR-A [41], McKay and Watson [44] an adapted version of the Kutcher’s Adolescent Depression Scale, Olsavsky et al. [35] the Children’s Depression Inventory (CDI; [57]), Rusow et al. [39] the Depression subscale of the BSI-18 [43], Thorne et al. [36] the Depression subscale of the Hospital Anxiety and Depression Scale (HADS; [58]). Price-Feeney et al. [49] assessed depressive symptoms using a single item based on the Youth Risk Behavior Surveillance System (YRBS; [59]), while Garthe et al. [45] employed a self-constructed item.

Eight studies reported measures of anxiety symptoms, seven of these studies compared non-binary and transgender individuals [35, 36, 38, 39, 46, 47, 52], with two of them additionally including cisgender individuals for comparison [38, 52]. One study compared non-binary and cisgender individuals [53]. Half of the studies measured anxiety symptoms with the Generalized Anxiety Disorder 7 (GAD-7; [60]) [46, 47, 52, 53]. Childs et al. [38] used the Anxiety subscale of the BASC-3 SPR-A [41], Olsavsky et al. [35] the Screen for Child Anxiety Related Emotional Disorders (SCARED; [61]), Rusow et al. [39] and Thorne et al. [36] the Anxiety subscales of the BSI-18 [43] and the HADS [58], respectively.

Self-harm rates were reported in seven studies, all of which presented data on non-binary and transgender youth [17, 18, 35, 36, 39, 47, 62]. However, only one study included additional data on cisgender youth for comparative analysis [39]. All included studies utilized a single-item assessment for self-harm, with four studies examining lifetime self-harming behavior [18, 36, 39, 62], three studies focusing on the past year [17, 35, 47] and one study assessing self-harm within the last three months [39].

Measurements related to suicidality were investigated in eleven studies. Ten of these studies compared non-binary individuals with transgender individuals [16–18, 35, 39, 45, 49, 50, 63, 64], with six studies additionally including comparisons with cisgender individuals [16, 45, 49, 50, 63, 64]. One study exclusively reported data on non-binary and cisgender individuals [65]. Suicidal ideation rates were evaluated in nine studies, with three reporting lifetime rates [16, 62, 65] and six reporting rates within the past year [17, 18, 35, 39, 45, 49]. Rates of suicide attempts were investigated in eight studies using a single item for the assessment, with five studies providing data on lifetime attempts [18, 50, 62, 64, 65] and three studies reporting attempt rates within the past year [17, 39, 49]. Individual studies have assessed additional aspects and/or timeframes related to suicidality. Each study has assessed passive death wish [62], past-year and lifetime suicide plan [39, 65], attempt plans [62], future suicide attempts [50], future suicides [18], and attempts requiring medical care [62], respectively.

### Quality assessment results

Details regarding the quality assessment of the included studies are presented in Additional file 1: Table S2. The risk of bias assessment using the Joanna and Briggs Institute appraisal tool (JBI) for Analytical Cross-Sectional Studies [29] provided following results: all studies provided sufficient details about the study subjects and the setting and employed appropriate statistical analysis methods. Most studies did not measure exposure. Approximately 86% of the articles used clear inclusion criteria and employed objective, standard criteria for measuring the condition, while about 81% ensured valid and reliable outcome measurement. In 71% of the included studies, confounding factors were identified, and strategies were implemented to address them. Thirteen studies were characterized by a high quality and a low risk of bias, whereas eight studies showed a moderate quality with a moderate risk of bias. None of the studies fell into the low-quality/high risk of bias category, indicating an overall high or acceptable quality across the studies.

**Table 1:** Characteristics of included studies

First author, year	Country	Sample (*n*) and groups	Population	Age (years)	Mental health outcomes	Mental health measures	Study quality
Aparicio-García et al. (2018) [16]	Spain	*N* = 782 *n* = 70 (9%) non-binary *n* = 180 (23%) transgender *n* = 532 (68%) cisgender	Participants were recruited through websites, Twitter, and different associations	14–25	General mental health	General Health Questionnaire (GHQ-12)	6
					Suicidal ideation lifetime	One item: “Once I have thought about suicide”	
Childs et al. (2022) [38]	USA	*N* = 426 *n* = 7 (1.6%) non-binary *n* = 10 (2.3%) transgender *n* = 272 (64.1%) cisgender female *n* = 137 (32.2%) cisgender male	Participants were enrolled in a psychiatric outpatient clinic	M = 14.94, SD = 1.5	General mental health	Behavior Assessment System for Children, Third Edition, Self-Report of Personality-Adolescent (BASC-3 SRP-A): Depression, Anxiety, Sense of inadequacy, Locus of control, Hyperactivity, Attention problems subscales	5
					Depressive symptoms	BASC-3 SRP-A: Depression subscale	
					Anxiety symptoms	BASC-3 SRP-A: Anxiety subscale	
Ciria-Barreiro et al. (2021) [37]	Spain	*N* = 1212 *n* = 213 non-binary *n* = 90 transgender *n* = 303 cisgender	Participants completed the Health Behaviour in School-aged Children (HBSC) study, recruited through schools	15–18	General mental health	HBSC Symptom Checklist: Psychological Complaints subscale	5
Clark et al. (2018) [17]	Canada	*N* = 839 *n* = 344 (41%) non-binary transgender *n* = 356 (42%) transgender male *n* = 139 (19%) transgender female	Participants completed the Canadian Trans Youth Health Survey, recruited through various methods	14–25	General mental health	One item: Self-reported mental health	6
					Self-harm past year	Non-suicidal self-injury past year	
					Suicidal ideation past year	Seriously considered suicide past year	
					Suicide attempt past year	Attempted suicide past year	
Garthe et al. (2022) [45]	USA	*N* = 4664 *n* = 1116 gender expansive (non-binary) *n* = 1116 transgender *n* = 1116 cisgender female *n* = 1116 cisgender male	Participants completed the Illinois Youth Survey (IYS), recruited through schools	Adolescent students in grades 8 to 12	Depressive symptoms	One item: “During the past 12 months did you ever feel so sad or hopeless almost every day for two weeks or more in a row that you stopped doing some usual activities?”	5
					Suicidal ideation past year	One item: “During the past 12 months, did you ever seriously consider attempting suicide?’’	
Jardas et al. (2023) [46]	USA	*N* = 1943 *n* = 640 (32.94%) non-binary AFAB *n* = 84 (4.32%) non-binary AMAB *n* = 990 (50.95%) transgender male *n* = 132 (6.79%) transgender female	Participants took part in the Gender Minority Youth (GMY) Study, recruited through Facebook and Instagram	14–18	Depressive symptoms	Center for Epidemiologic Studies Depression Scale (CES-D)	7
					Anxiety symptoms	Generalized Anxiety Disorder-7 Scale (GAD-7)	
Kaltiala et al. (2023) [53]	Finland	*N* = 130,322 *n* = 4094 non-binary/other *n* = 813 opposite sex identification (transgender) *n* = 124,219 cisgender	Participants completed the School Health Promotion Study by the National Institute for Health and Welfare, recruited through schools	13–20	Depressive symptoms	Patient Health Questionnaire (PHQ-2)	6
					Anxiety symptoms	Generalized Anxiety Disorder-7 Scale (GAD-7)	
McKay & Watson (2020) [44]	USA	*N* = 17,112 *n* = 2362 (65.2%) non-binary AFAB *n* = 237 (6.5%) non-binary AMAB *n* = 903 (24.9%) transgender male *n* = 122 (3.4%) transgender female *n* = 4715 (44.5%) cisgender female *n* = 2335 (22.0%) cisgender male	Participants completed the LGBTQ National Teen Survey, recruited through various methods	13–17	Depressive symptoms	Kutcher’s Adolescent Depression Scale	7
Meyer et al. (2021) [65]	USA	*N* = 1507 *N* = 664 (18–24 years) *n* = 66 (9.9%) non-binary *n* = 390 (58.7%) cisgender female *n* = 208 (31.4%) cisgender male	Participants took part in the Generations Study for sexual minority people, recruited through the Gallup Daily Tracking Survey	18–59 (18–24; 34–41; 52–59)	Suicidal ideation lifetime	Army Study to Assess Risk and Resilience in Service Members instrument	7
					Suicide plan lifetime	Army Study to Assess Risk and Resilience in Service Members instrument	
					Suicide attempt lifetime	Army Study to Assess Risk and Resilience in Service Members instrument	
Olsavsky et al. (2023) [35]	USA	*N* = 75 *n* = 6 non-binary *n* = 41 transgender male *n* = 28 transgender female	Participants were recruited at a gender-affirming multidisciplinary clinic	11–18	Depressive symptoms	Children’s Depression Inventory (CDI)	8
					Anxiety symptoms	Screen for Child Anxiety Related Emotional Disorders (SCARED)	
					Self-harm past year	One item based on the Suicide Behaviors Questionnaire-Revised (SBQ-R) and Columbia Suicide Severity Rating Scale (C-SSRS)	
					Suicidal ideation past year	One item from the SBQ-R	
Parodi et al. (2022) [47]	USA	*N* = 252 *n* = 70 non-binary AFAB *n* = 59 non-binary AMAB *n* = 66 transgender male *n* = 57 transgender female	Participants took part in the Project Advancing Voices of Adolescents identifying as Nonbinary and Transgender (Project AVANT), recruited through various methods	14–18	Depressive symptoms	Patient Health Questionnaire (PHQ-2)	7
					Anxiety symptoms	Generalized Anxiety Disorder-7 Scale (GAD-7)	
					Self-harm past year	One item of the Youth Risk Behavior Survey	
Peng et al. (2019) [48]	China	*N* = 385 *n* = 109 non-binary *n* = 109 transgender male *n* = 167 transgender female	Participants completed an online survey for transgender and gender-diverse people, recruited through various methods	12–18	Depressive symptoms	Center for Epidemiological Studies Depression 9-item (CES-D-9)	7
					Anxiety symptoms	Not reported separately by gender identity.	
					Suicidal ideation	Not reported separately by gender identity.	
Price-Feeney et al. (2020) [49]	USA	*N* = 25,396 *n* = 3797 non-binary AFAB *n* = 957 non-binary AMAB *n* = 3103 transgender male *n* = 508 transgender female *n* = 8073 cisgender female *n* = 8954 cisgender male	Participants took part in a study for cisgender LGBQ, transgender and non-binary youth, recruited through Facebook and Instagram	13–24	Depressive symptoms	One item from the Youth Risk Behavior Surveillance System (YRBS)	6
					Suicidal ideation past year	One item from the YRBS: “During the past 12 months, did you ever seriously consider attempting suicide?”	
					Suicide attempt past year	One item from the YRBS: “During the past 12 months, how many times did you actually attempt suicide?”	
Rimes et al. (2017) [18]	UK	*N* = 677 *n* = 269 non-binary AFAB *n* = 93 non-binary AMAB *n* = 210 transgender male *n* = 105 transgender female	Participants were LGBTQ young adults, who took part in the “Youth Chances” project, recruited through various methods	16–25	General mental health	One item: “Do you have any health conditions or illnesses which affect you and interfere with your normal activities?”	4
					Self-harm lifetime	One item: “Have you ever hurt yourself on purpose? This is sometimes called ‘self-harm’.”	
					Suicidal ideation past year	One item from the SBQ-R	
					Suicide attempt lifetime	One item from the SBQ-R	
					Suicide in the future	One item from the SBQ-R	
Rusow et al. (2022) [39]	USA	*N* = 108 *n* = 70 non-binary transgender and gender diverse *n* = 38 binary transgender and gender diverse	Participants took part in the Trans Youth of Color Study (TRUTH), recruited through various methods	16–24	General mental health	18-item Brief Symptom Inventory (BSI): Depression, Anxiety and Somatic Complaints subscales	5
					Depressive symptoms	BSI: Depression Subscale	
					Anxiety symptoms	BSI: Anxiety Subscale	
					Self-harm in the last three month	One item from the BSI	
					Self-harm lifetime	One item from the BSI	
					Suicidal ideation past year	One item from the BSI	
					Suicide plan past year	One item from the BSI	
					Suicide attempt past year	One item from the BSI	
Srivastava et al. (2021) [50]	USA	*N* = 592 *n* = 141 (23.9%) non-binary *n* = 120 (20.4%) transgender *n* = 328 (55.7%) cisgender	Participants were recruited from The Trevor Project, a gender- and sexual minority youth-focused suicide crisis prevention service provider	12–24	Depressive symptoms	Center for Epidemiologic Studies Depression Scale Short Form (CES-D-4)	7
					Suicide attempt lifetime	One item from the Columbia-Suicide Severity Rating Scale (C-SSRS)	
					Suicide attempt in the future	One item from the Columbia-Suicide Severity Rating Scale (C-SSRS)	
Sterzing et al. (2017) [51]	USA	*N* = 1177 *n* = 189 genderqueer (non-binary) AFAB *n* = 52 genderqueer male (non-binary) AMAB *n* = 47 transgender male *n* = 19 transgender female *n* = 478 cisgender female *n* = 389 cisgender male	Participants were sexual and gender minority youth, recruited through Facebook, community organizations and adverts	14–19	Depressive symptoms	Center for Epidemiological Studies Short Depression Scale (CESD-10)	4
Thoma et al. (2019) [62]	USA	*N* = 2020 *n* = 375 non-binary AFAB *n* = 43 non-binary AMAB *n* = 616 transgender male *n* = 63 transgender female *n* = 654 cisgender female *n* = 218 cisgender male *n* = 51 questioning	Participants completed an online survey for transgender and cisgender adolescents, recruited through Facebook and Instagram	14–18	Passive death wish lifetime	Item adapted from the Youth Risk Behavior Survey and the Columbia–Suicide Severity Rating Scale	7
					Self-harm lifetime	Item adapted from the Youth Risk Behavior Survey and the Columbia–Suicide Severity Rating Scale	
					Suicidal ideation lifetime	Item adapted from the Youth Risk Behavior Survey and the Columbia–Suicide Severity Rating Scale	
					Suicide attempt plan lifetime	Item adapted from the Youth Risk Behavior Survey and the Columbia–Suicide Severity Rating Scale	
					Suicide attempt lifetime	Item adapted from the Youth Risk Behavior Survey and the Columbia–Suicide Severity Rating Scale	
					Suicide attempt lifetime requiring medical care	Item adapted from the Youth Risk Behavior Survey and the Columbia–Suicide Severity Rating Scale	
Thorne et al. (2018) [36]	UK	*N* = 388 *n* = 57 (14.7%) non-binary transgender *n* = 331 (85.3%) binary transgender	Participants were referred for an assessment at a national transgender health service and recruited through the clinic	16–25	Depressive symptoms	Hospital anxiety and depression scale (HADS)	5
					Anxiety symptoms	Hospital anxiety and depression scale (HADS)	
					Self-harm lifetime	Non-suicidal self-injury (NSSI) questionnaire - treatment related (SIQ-TR)	
Toomey al. (2018) [64]	USA	*N* = 120,617 *n* = 344 (0.3%) non-binary *n* = 175 (0.1%) transgender AFAB *n* = 202 (0.2%) transgender AMAB *n* = 60,973 (50.6%) cisgender female *n* = 57,871(48%) cisgender male *n* = 1052 (0.9%) questioning	Participants completed the Profiles of Student Life: Attitudes and Behaviors survey, data was collected by the Search Institute via community partnerships	11–19	Suicide attempt lifetime	One item: “Have you ever tried to kill yourself?”	5
Wang et al. (2020) [52]	China	N = 12,108 *n* = 112 (2.0%) non-binary AFAB *n* = 138 (2.1%) non-binary AMAB *n* = 861 (15.4%) transgender male *n* = 208 (3.2%) transgender female *n* = 4142 (74.1%) cisgender female *n* = 5855 (89.8%) cisgender male *n* = 475 (8.5%) questioning AFAB *n* = 317 (4.9%) questioning AMAB	Participants were students attending public secondary schools (grades 7–11), recruited through schools	M = 15.8, SD = 1.0	Depressive symptoms	PHQ-9	7
					Anxiety symptoms	GAD-7	

### General mental health problems

**Fig. 2:**
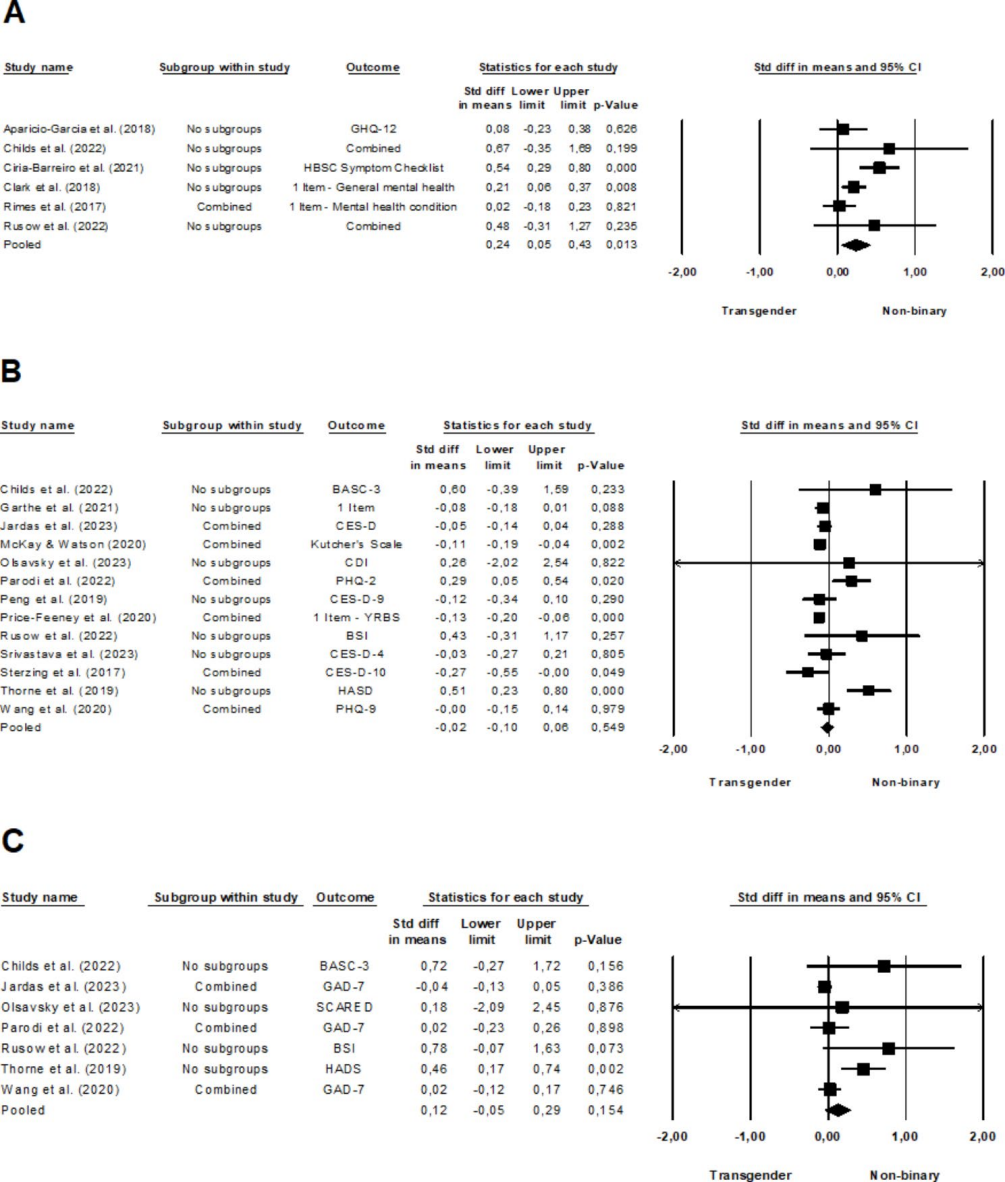
Forest plot comparing non-binary and transgender youth regarding their **A** general mental health, **B** depressive and **C** anxiety symptoms. A larger effect size indicates a worse mental health outcome in non-binary individuals.

**Fig. 3:**
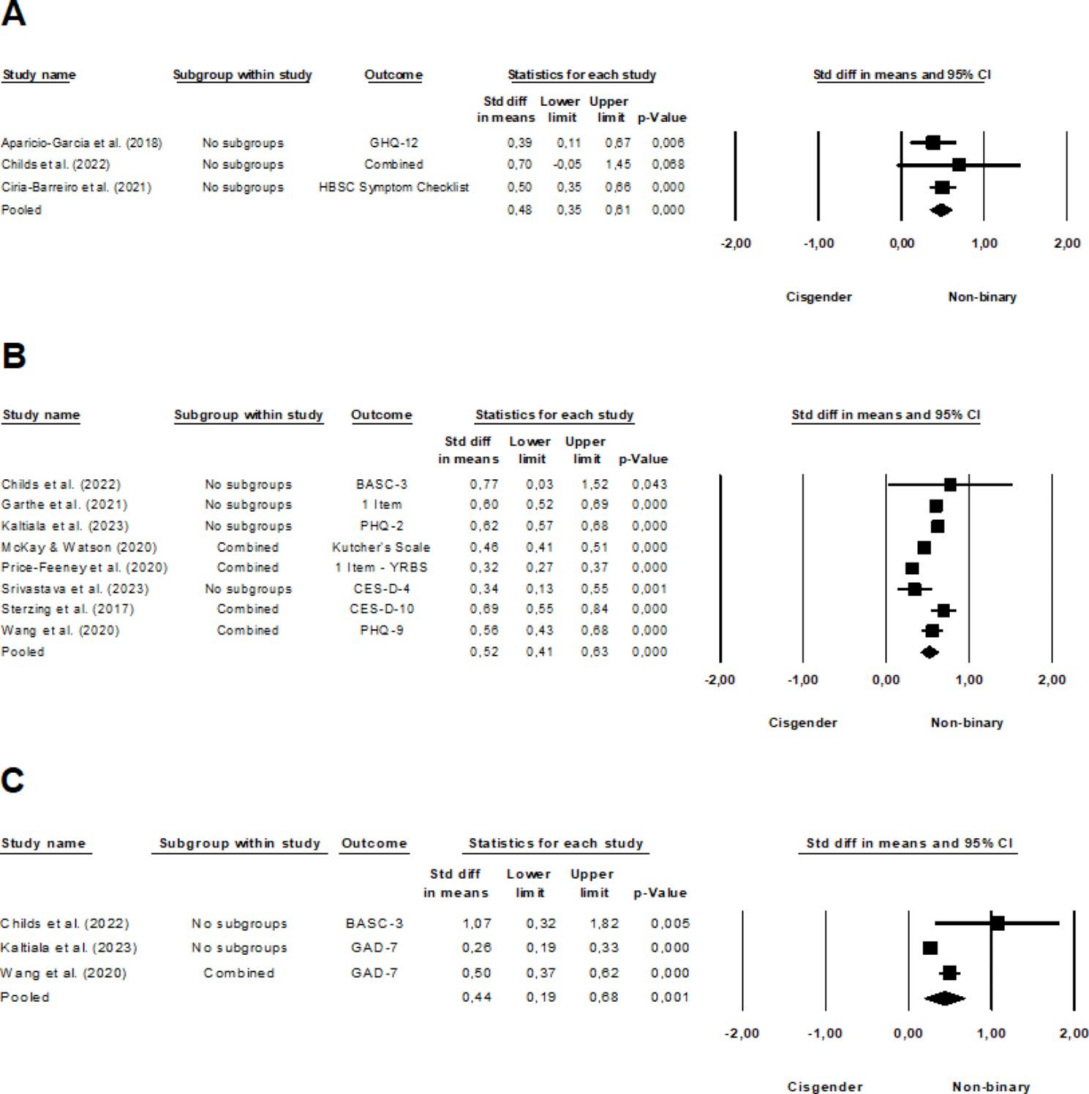
Forest plot comparing non-binary and cisgender youth regarding their **A** general mental health, **B** depressive and **C** anxiety symptoms. A larger effect size indicates a worse mental health outcome in non-binary individuals.

The meta-analysis of six studies [16–18, 37–39] comparing non-binary and transgender youth revealed a significant, yet small effect (*d* = 0.24, 95% CI, 0.05–0.43, *p* =.013), with moderate heterogeneity among the studies (*I*^2^ = 56.77%, *Q*(5) = 11.56, *p* =.041). Non-binary youth reported poorer general mental health than their transgender peers. The forest plot in Fig. 2a illustrates these findings. When comparing non-binary and cisgender youth using data from three studies [16, 17, 37], a significant, almost moderate effect was observed (*d* = 0.48, 95% CI, 0.35–0.61, *p* <.001), with no significant heterogeneity among the studies (*I*^2^ = 0.00%, *Q*(2) = 0.82, *p* =.662). Non-binary youth reported a more impaired general mental health compared to cisgender youth, as depicted in the forest plot (Fig. 3a).

### Depressive symptoms

The analysis of thirteen studies [35, 36, 38, 39, 44–52] comparing non-binary and transgender individuals in terms of depressive symptoms did not reveal a significant effect (*d* = -0.02, 95% CI, -0.10-0.06, *p* =.549), see also Fig. 2b. There was moderate heterogeneity among the studies (*I*^*2*^ = 66.49%, *Q*(12) = 35.81, *p* <.001). In the pooled analysis of eight studies [38, 44, 45, 49–53] comparing non-binary and cisgender individuals on depressive symptoms, a moderate effect was observed (*d* = 0.52, 95% CI, 0.41–0.63, *p* <.001), with high heterogeneity among the studies (*I*^*2*^ = 92.04%, *Q*(7) = 87.97, *p* <.001). Non-binary individuals reported more depressive symptoms compared to cisgender individuals, as presented in the forest plot in Fig. 3b.

### Anxiety symptoms

Seven studies were pooled examining non-binary and transgender individuals in relation to anxiety symptoms [35, 36, 38, 39, 46, 47, 52]. The analysis did not yield a significant effect (*d* = 0.12, 95% CI, -0.05-0.29, *p* =.154), with moderate heterogeneity observed among the studies (*I*^*2*^ = 61.94%, *Q*(6) = 15.77, *p* =.015). For a detailed graphical representation of these outcomes see Fig. 2c. By pooling of three studies that compared non-binary and cisgender individuals on anxiety symptoms [38, 52, 53], a small effect was detected (*d* = 0.44, 95% CI, 0.19–0.68, *p* =.001), accompanied by high heterogeneity among the studies (*I*^*2*^ = 85.81%, *Q*(2) = 14.10, *p* =.001). Across studies, non-binary individuals were found to experience more anxiety symptoms than their cisgender counterparts, as illustrated in the forest plot (Fig. 3c).

### Self-harm rates

**Fig. 4:**
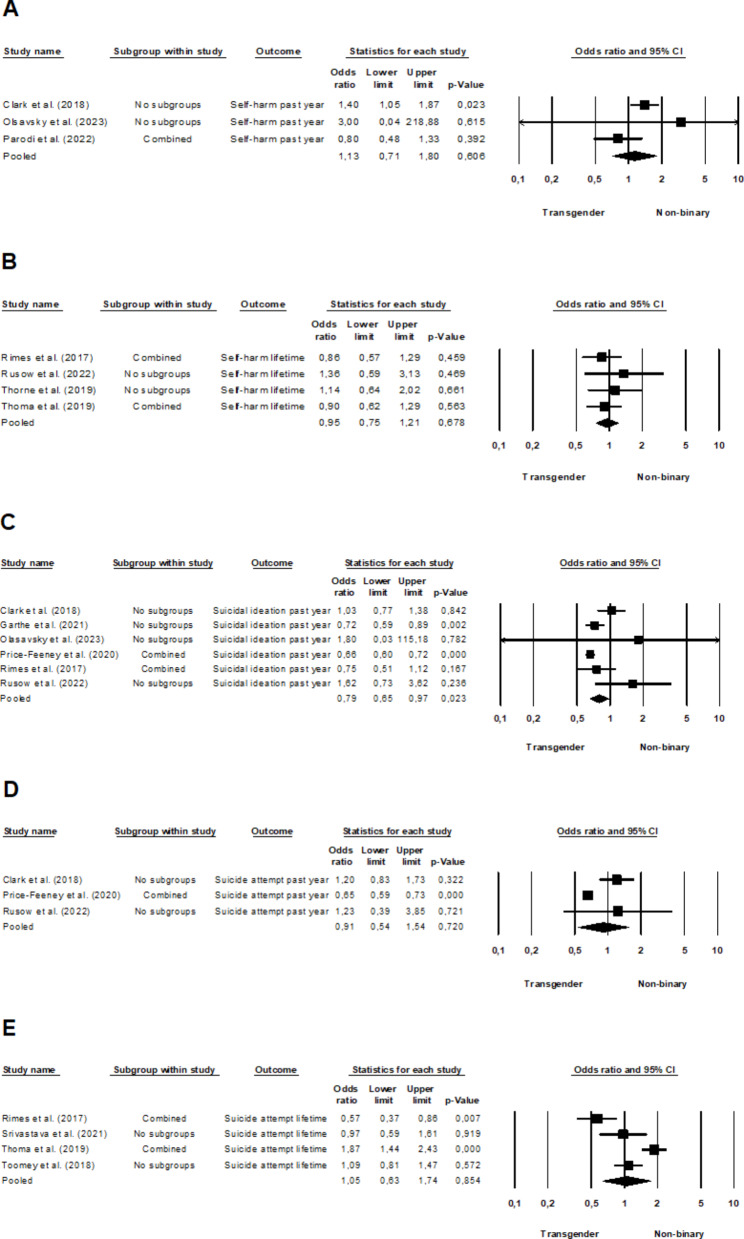
Forest plot comparing non-binary and transgender youth regarding **A** past year and **B** lifetime self-harm rates, as well as **C** past year suicidal ideation, **D** past year and **E** lifetime suicide attempt rates. A larger effect size indicates a worse mental health outcome in non-binary individuals.

The pooled analysis of three studies [17, 35, 47] including data on self-harm rates of the past year regarding non-binary and transgender youth revealed a non-significant effect (OR = 1.13, 95% CI, 0.71–1.80, *p* =.606), while the heterogeneity was moderate (*I*^*2*^ = 45.15%, *Q*(2) = 3.65, *p* =.162). Similarly, the pooling of lifetime prevalence rates from four studies [18, 36, 39, 62] revealed no significant effect (OR = 0.95, 95% CI, 0.75–1.21, *p* =.678), with no heterogeneity among the studies (*I*^*2*^ = 0.00%, *Q*(3) = 1.43, *p* =.699). For the corresponding forest plots see Fig. 4a and b, respectively.

### Suicidality rates

The meta-analysis of six studies [17, 18, 35, 39, 45, 49] exploring suicidal ideation in the past year among non-binary and transgender individuals revealed a statistically significant effect (OR = 0.79, 95% CI, 0.65–0.97, *p* =.023), with moderate heterogeneity (*I*^*2*^ = 61.39%, *Q*(5) = 12.95, *p* =.024). Notably, non-binary individuals demonstrated a lower prevalence of suicidal ideation than transgender individuals within the past year, for the corresponding forest plot see Fig. 4c. Additionally, when pooling three studies [17, 39, 49] that compared non-binary and transgender individuals in terms of past year suicide attempts, the results indicated no significant effect (OR = 0.91, 95% CI, 0.54–1.54, *p* =.720). High heterogeneity was observed among the studies (*I*^*2*^ = 81.60%, *Q*(2) = 10.87, *p* =.004). Finally, an analysis of four studies [18, 50, 62, 64] encompassing non-binary and transgender youth concerning lifetime suicide attempts showed no significant effect (OR = 1.05, 95% CI, 0.63–1.74, *p* =.854) and high heterogeneity (*I*^2^ = 87.89%, *Q*(3) = 24.77, *p* <.001). Forest plots including the results on suicide attempt rates are presented in Fig. 4d and e.

**Fig. 5:**
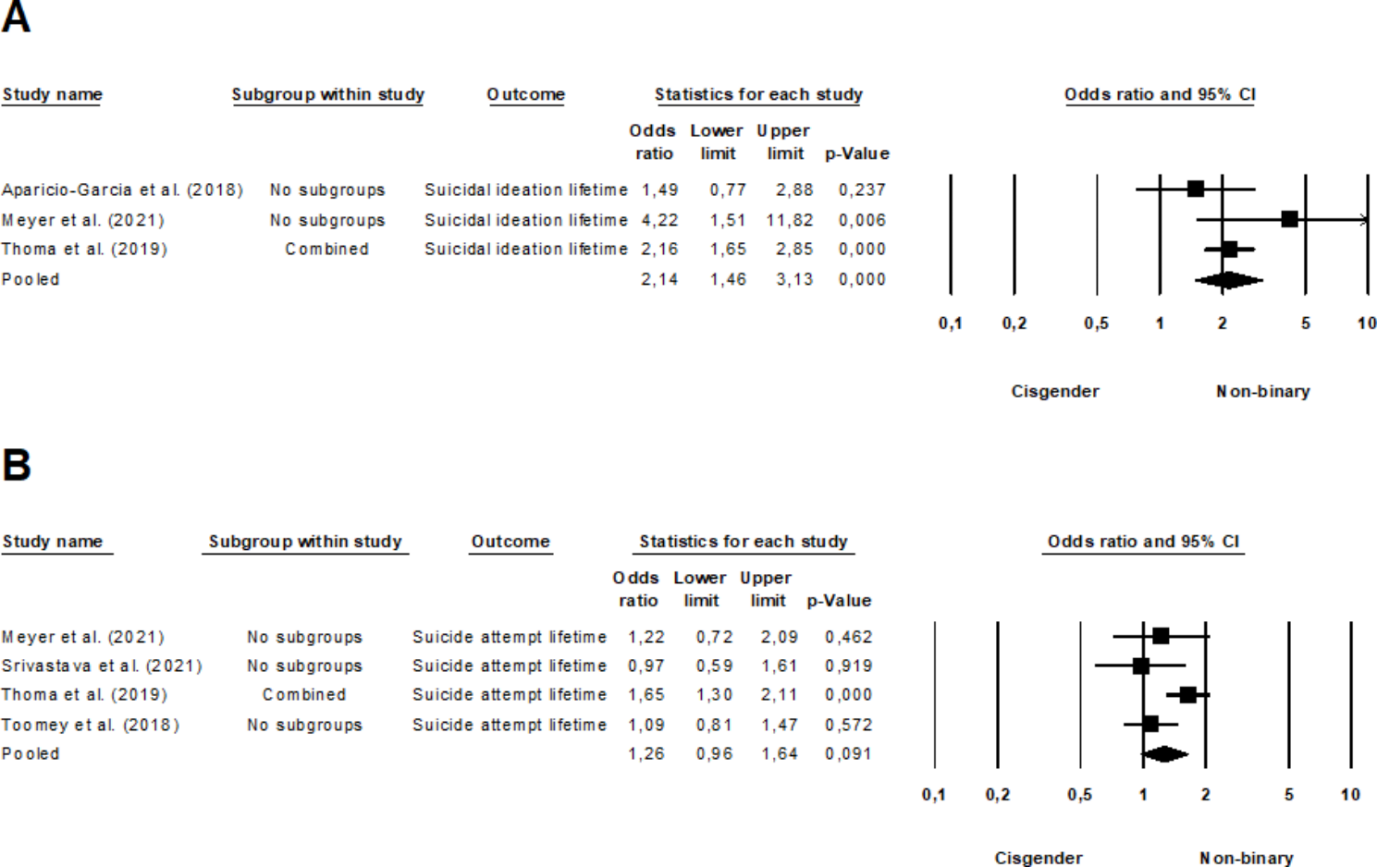
Forest plot comparing non-binary and cisgender youth regarding **A** lifetime suicidal ideation and **B** suicide attempt rates. A larger effect size indicates a worse mental health outcome in non-binary individuals.

Pooling three studies [16, 62, 65] that analyzed lifetime suicidal ideation among non-binary and cisgender youth revealed a statistically significant effect (OR = 2.14, 95% CI, 1.46–3.13, *p* <.001), with moderate heterogeneity (*I*^*2*^ = 29.50%, *Q*(2) = 2.84, *p* =.242). This implies that non-binary youth exhibit a higher lifetime prevalence of suicidal ideation than their cisgender peers (Fig. 5a). On the other hand, an analysis of four studies [50, 62, 64, 65] encompassing non-binary and cisgender youth concerning lifetime suicide attempts showed no significant effect (OR = 1.26, 95% CI, 0.96–1.64, *p* =.091), while heterogeneity was moderate (*I*^*2*^ = 52.96%, *Q*(3) = 6.38, *p* =.095). For an in-depth illustration of these findings, see Fig. 5b.

### Sensitivity analysis

In the sensitivity analysis conducted for the general mental health meta-analyses, two studies [38, 39], where outcome measures were combined from subscales of the same instrument, were excluded. However, this exclusion did not impact the overall significance of the results. We also re-analyzed the results regarding depressive symptoms without the two studies that used a single item instead of a standardized measure [45, 49]. Again, the analysis did not alter our findings in either the comparison of non-binary and transgender youth or non-binary and cisgender youth. A sensitivity analysis was performed for lifetime self-harm rates, excluding the study by Rimes et al. [18], which assessed the broader variable of self-harm rather than non-suicidal self-injury (NSSI) like the other three studies. Importantly, this exclusion did not alter the significance of the results. To evaluate potential sampling bias in the meta-analysis regarding lifetime suicidal ideation and attempt rates comparing non-binary and cisgender youth, we re-analyzed the data without the study of Meyer et al. [65], which included only young adult participants. However, the exclusion of this study also did not alter the results significantly. Due to the limited number of studies included in the meta-analyses, it was not appropriate to use funnel plots to assess publication bias and perform meta-regressions to account for covariates and investigate the reasons for heterogeneity between the included studies [66].

## Discussion

To the best of our knowledge, this is the first meta-analysis and systematic review focusing on the mental health of non-binary youth. In this study, non-binary youth were compared to their transgender and cisgender counterparts in terms of general mental health, depressive and anxiety symptoms, self-harm, and suicidal behavior. A total of 21 studies were included in the meta-analysis, from which 13 distinct analyses were conducted, focusing on the specific mental health outcomes and group comparisons. Originating from six different countries, the included studies comprised a diverse sample of 16,114 non-binary, 11,925 transgender, and 283,278 cisgender youth aged between 11 and 25 years.

Our results showed that non-binary youth reported poorer general mental health than transgender youth, with a small but statistically significant effect size. Compared to cisgender youth, non-binary individuals had significantly more impaired general mental health, the results indicating an almost moderate effect. Our findings align with the systematic review by Chew et al. [27] on overall mental health and the study by de Graaf et al. [26] on psychological difficulties in non-binary individuals. Depressive symptoms comparisons between non-binary and transgender individuals revealed no significant effect. In contrast, non-binary youth showed more depressive symptoms than cisgender peers, with a moderate effect size. A similar pattern emerged for anxiety, with no significant differences between non-binary and transgender youth, but a small effect suggesting more anxiety symptoms in non-binary youth compared to cisgender individuals. However, Chew et al. [27], based on one study [36], reported significantly higher levels of anxiety and depression in non-binary adolescents compared to binary transgender youth, whereas our meta-analysis found no significant differences between these groups. This does not imply that non-binary individuals are unaffected, as both non-binary and transgender youth generally exhibit higher levels of anxiety and depression compared to cisgender individuals [67–70]. Thus, the similar rates between non-binary and transgender youth in our analysis suggest a greater impact on non-binary youth relative to cisgender peers. The analysis of self-harm rates for non-binary and transgender youth over the past year showed no significant effect, as did the synthesis of lifetime prevalence rates. Although Chew et al. [27] reported mixed evidence on self-harm, our quantitative synthesis, which included the two studies [17, 36] from Chew et al.’s review, found no significant difference between non-binary and transgender individuals. Our analysis of suicidal ideation revealed a significant effect, with non-binary youth reporting lower rates than transgender individuals. However, no significant effects were found for suicide attempts, whether for past year or lifetime data. Non-binary youth exhibited a significantly higher lifetime prevalence of suicidal ideation compared to cisgender youth, though no significant effect was observed in lifetime suicide attempts. Similarly, Chew et al. [27] presented data from Aparicio-Garcia et al. [16], showing higher lifetime suicidal ideation in non-binary youth compared to cisgender youth, a finding consistent with our results.

The observed mental health disparities regarding the general mental health, depressive, and anxiety symptoms, self-harm and suicidality may be attributed to factors that generally affect individuals of gender minorities, such as experiences of stigma, discrimination, victimization, and non-affirmation discussed within the minority stress framework [23, 25, 71, 72]. Continuous exposure to these stressors, particularly among non-binary youth experiencing gender dysphoria [73], may lead to dysregulation of the hypothalamic-pituitary-adrenal (HPA) axis, which regulates the body’s stress response. DuBois et al. [74] found that higher levels of enacted stigma in transgender and gender diverse individuals were linked to a blunted cortisol awakening response and a slower daily decline in cortisol levels, indicating chronic stress response. Prolonged activation of the stress response system may disrupt coping mechanisms, thereby increasing the risk of negative mental health outcomes among non-binary individuals [73].

Specific factors contributing to the poorer mental health of non-binary youth include a lack of understanding, limited visibility, and the invalidation of non-binary identities [1, 26, 75], coupled with distinctive features in the identity development of non-binary people [1]. Additionally, a unique experience of gender dysphoria specific to non-binary individuals, as suggested by qualitative studies [76, 77], may further shape these disparities. Furthermore, barriers to (gender-affirming) health care may also play a role in contributing to the impaired mental health of non-binary youth [3, 7, 27]. These challenges may be further exacerbated by intersecting factors, including general mental health problems and depression, as well as various unfavorable social factors, such as traumatic experiences (e.g., physical or sexual abuse, relationship violence, bullying victimization), familial factors (e.g., less parent connectedness, running away from home, homelessness due to being cast out and rejected by parents), and school-related factors (e.g., lower grades, lower levels of perceived school safety), all of which may lead to the elevated risk of self-harm and suicidal behavior among non-binary youth, similar to transgender youth [78–80].

This meta-analysis has significant implications for both policy and clinical practice. The factors contributing to mental health disparities in non-binary youth are multifaceted, encompassing issues such as a lack of understanding, legal and social recognition, limited visibility, and invalidation of non-binary identities [1, 3, 75]. The marginalization and denial of non-binary identities also result in the lack of research in many regions, often driven by societal stigma and legal restrictions. In some countries, non-binary gender expression may face criminalization, further inhibiting support for this population [81]. From a policy perspective, efforts should focus on enhancing awareness and education about non-binary identities in various sectors, including education, healthcare, and social services. Additionally, sociopolitical interventions, such as addressing systemic inequalities and advocating for broader social reforms, are necessary alongside policies to ensure legal recognition and protection of non-binary individuals, promoting inclusivity and reducing societal stigmatization. Creating safe spaces and support networks is crucial to validate non-binary identities and foster a sense of belonging. Distinctive features in the identity development of non-binary individuals, a unique experience of gender dysphoria specific to this group, and further mental health concerns may contribute to these challenges [1, 25, 76, 77, 82]. A study by Conlin et al. [82] suggests that some non-binary individuals become aware of their identities early in life, while others discover them in adolescence or adulthood, often triggered by learning about non-binary identities. Unlike linear models of identity development, non-binary individuals frequently experience fluidity in their identities, navigating between stability and change, with fluidity sometimes limited by personal or societal factors [1]. According to Paz Galupo et al. [76], non-binary individuals experience dysphoria in a more fluid and dynamic manner, often challenging conventional medical approaches designed for binary transgender individuals. Our study emphasizes the necessity of gender-affirming health care in cases where non-binary youth are experiencing GI/GD [3, 7], with careful consideration of their identity stability within the non-binary spectrum, especially when discussing irreversible gender-affirming medical interventions. Given the elevated rates of severe psychiatric symptoms, such as self-harm and suicidality, specific screening and targeted interventions addressing these specific challenges are crucial for effective clinical practice and policy initiatives. Psychotherapeutic approaches, including both individual and family-based interventions, are essential, as family acceptance is associated with higher self-esteem, increased social support, and better overall health, while also reducing the risk of depression, substance abuse, and suicidality [83].

### Strengths and limitations

This study stands out as the most comprehensive analysis to date, offering a synthesis of data on the mental health of non-binary youth. Our meta-analysis covers a broad spectrum of mental health outcomes, including general mental health, depressive and anxiety symptoms, self-harm, and suicidality, providing an overview of the mental health of non-binary youth. Additionally, the study includes moderate to high-quality studies, contributing to the robustness of the findings. Methodologically, the study is characterized by thorough adherence to relevant guidelines and a sensitivity analysis regarding studies prone to bias, ensuring transparency and validity.

Despite its strengths, this study has certain limitations. The study encompassed a range of mental health outcomes; however, the limited number of studies available for certain sub-analyses could impact the generalizability of the findings. The need to conduct separate analyses due to variations in group comparisons (some studies included all three groups, while others only compared two) also limits the comprehensiveness of the findings. Furthermore, the studies in this meta-analysis employed different assessments of gender identity and lacked information on perceived gender dysphoria, as well as information about psychological and medical interventions received by non-binary youth in the sample. Additionally, variations in outcome measures across studies contribute to the complexity of interpreting the findings. The reliance on self-reported data raises the possibility of response and recall bias. These limitations underscore the need for caution in generalizing the results and highlight areas for future research to address gaps in our understanding of non-binary youth’s mental health.

Future research in the field of non-binary youth’s mental health should prioritize the standardization of the assessment of non-binary identity as a distinct category, considering its multifaceted nature and potentially diverse subcategories. Adopting a consistent analysis based on sex assigned at birth could be essential in revealing specific mental health challenges that may vary across the different assigned sexes. Moreover, examining GI/GD and the effectiveness of psychological and medical interventions on mental health in non-binary individuals could contribute valuable insights. Lastly, the inclusion of diverse samples from various cultural contexts is crucial for understanding the intersectionality of non-binary individuals’ mental health and tailoring interventions to meet their specific needs.

## Conclusion

This systematic review and meta-analysis showed that non-binary youth experience poorer general mental health compared to both transgender and cisgender counterparts. While they exhibit comparable levels of depressive and anxiety symptoms to transgender individuals, they demonstrate higher levels than their cisgender peers. Our findings revealed that self-harm and suicidal behavior patterns are similar between non-binary and transgender youth in certain aspects and time frames. Clinically, this underscores the critical need for targeted mental health interventions for non-binary youth and highlights the urgency of gender-affirming mental health support, while policy efforts should focus on creating inclusive frameworks. Future research should standardize the assessment of non-binary identity, considering sex assigned at birth, to uncover nuanced mental health aspects within this diverse population. The comprehensive insights provided by this study lay the groundwork for informed decisions in clinical, policy, and research domains.

## Electronic supplementary material

Supplementary Material 1

## Data Availability

The datasets used and/or analyzed during the current study are available from the corresponding author on reasonable request.

## References

[1] Matsuno E, Budge SL. Non-binary/Genderqueer identities: a critical review of the literature. Curr Sex Health Rep. 2017;9:116–20.

[2] Lee JY, Rosenthal SM. Gender-affirming care of transgender and gender-diverse youth: current concepts. Annu Rev Med. 2023;74:107–16. 36260812 10.1146/annurev-med-043021-032007 PMC11045042

[3] Coleman E, Radix AE, Bouman WP, Brown GR, de Vries ALC, Deutsch MB, et al. Standards of Care for the health of transgender and gender diverse people, Version 8. Int J Transgend Health. 2022;23:S1–259. 36238954 10.1080/26895269.2022.2100644 PMC9553112

[4] American Psychological Association. Guidelines for psychological practice with transgender and gender nonconforming people. Am Psychol. 2015;70:832–64. 26653312 10.1037/a0039906

[5] World Health Organization. ICD-11 Clinical Descriptions and Diagnostic Guidelines for Mental and Behavioural Disorders. 2023. https://icd.who.int/browse11/l-m/en. Accessed 17 Jun 2023.

[6] American Psychiatric Association. Diagnostic and statistical manual of mental disorders. In: Text revision (DSM-V-TR). 5th ed. Arlington: American Psychiatric Publishing; 2022.

[7] Hodax JK, DiVall S. Gender-affirming endocrine care for youth with a nonbinary gender identity. Ther Adv Endocrinol Metab. 2023. 10.1177/20420188231160405. 37006780 10.1177/20420188231160405 PMC10064168

[8] Spizzirri G, Eufrásio R, Lima MCP, de Carvalho Nunes HR, Kreukels BPC, Steensma TD, et al. Proportion of people identified as transgender and non-binary gender in Brazil. Sci Rep. 2021;11:1–7. 33500432 10.1038/s41598-021-81411-4 PMC7838397

[9] Van Caenegem E, Wierckx K, Elaut E, Buysse A, Dewaele A, Van Nieuwerburgh F, et al. Prevalence of gender nonconformity in Flanders, Belgium. Arch Sex Behav. 2015;44:1281–7. 25588709 10.1007/s10508-014-0452-6

[10] Kuyper L, Wijsen C. Gender identities and gender dysphoria in the Netherlands. Arch Sex Behav. 2014;43:377–85. 23857516 10.1007/s10508-013-0140-y

[11] Reisner SL, Hughto JMW. Comparing the health of non-binary and binary transgender adults in a statewide non-probability sample. PLoS ONE. 2019;14:e0221583. 31454395 10.1371/journal.pone.0221583 PMC6711503

[12] Scheim AI, Coleman T, Lachowsky N, Bauer GR. Health care access among transgender and nonbinary people in Canada, 2019: a cross-sectional survey. Can Med Association Open Access J. 2021;9:E1213–22. 10.9778/cmajo.20210061 PMC8695530 34933879

[13] James HA, Chang AY, Imhof RL, Sahoo A, Montenegro MM, Imhof NR, et al. A community-based study of demographics, medical and psychiatric conditions, and gender dysphoria/incongruence treatment in transgender/gender diverse individuals. Biol Sex Differ. 2020;11:1–10. 33023634 10.1186/s13293-020-00332-5 PMC7539507

[14] Cheung AS, Leemaqz SY, Wong JWP, Chew D, Ooi O, Cundill P, et al. Non-binary and binary gender identity in Australian trans and gender diverse individuals. Arch Sex Behav. 2020;49:2673–81. 32285311 10.1007/s10508-020-01689-9

[15] Kidd KM, Sequeira GM, Douglas C, Paglisotti T, Inwards-Breland DJ, Miller E et al. Prevalence of gender-diverse youth in an urban school district. Pediatrics. 2021;147. 10.1542/peds.2020-049823 PMC8168604 34006616

[16] Aparicio-García ME, Díaz-Ramiro EM, Rubio-Valdehita S, López-Núñez MI, García-Nieto I. Health and well-being of Cisgender, Transgender and non-binary young people. Int J Environ Res Public Health. 2018;15:2133. 30274141 10.3390/ijerph15102133 PMC6209926

[17] Clark BA, Veale JF, Townsend M, Frohard-Dourlent H, Saewyc E. Non-binary youth: access to gender-affirming primary health care. Int J Transgenderism. 2018;19:158–69.

[18] Rimes KA, Goodship N, Ussher G, Baker D, West E. Non-binary and binary transgender youth: comparison of mental health, self-harm, suicidality, substance use and victimization experiences. Int J Transgenderism. 2017;0. 10.1080/15532739.2017.1370627 PMC6831005 32999609

[19] Handler T, Hojilla JC, Varghese R, Wellenstein W, Satre DD, Zaritsky E. Trends in referrals to a pediatric transgender clinic. Pediatrics. 2019;144:20191368. 10.1542/peds.2019-1368 PMC6855897 31619510

[20] Twist J, de Graaf NM. Gender diversity and non-binary presentations in young people attending the United Kingdom’s national gender Identity Development Service. Clin Child Psychol Psychiatry. 2019;24:277–90. 30326742 10.1177/1359104518804311

[21] Mirabella M, Piras I, Fortunato A, Fisher AD, Lingiardi V, Mosconi M et al. Gender identity and non-binary presentations in adolescents attending two Specialized services in Italy. 2022. 10.1016/j.jsxm.2022.03.215 10.1016/j.jsxm.2022.03.215 35370103

[22] Chen D, Abrams M, Clark L, Ehrensaft D, Tishelman AC, Chan YM, et al. Psychosocial characteristics of Transgender Youth seeking gender-affirming Medical Treatment: baseline findings from the Trans Youth Care Study. J Adolesc Health. 2021;68:1104–11. 32839079 10.1016/j.jadohealth.2020.07.033 PMC7897328

[23] Chodzen G, Hidalgo MA, Chen D, Garofalo R. Minority stress factors Associated with Depression and anxiety among transgender and gender-nonconforming youth. J Adolesc Health. 2019;64:467–71. 30241721 10.1016/j.jadohealth.2018.07.006 PMC6528476

[24] Van Donge N, Schvey NA, Roberts TA, Klein DA. Transgender dependent adolescents in the U.S. military health care system: demographics, treatments sought, and health care service utilization. Mil Med. 2019;184:e447–54. 30325452 10.1093/milmed/usy264

[25] Wittlin NM, Kuper LE, Olson KR. Mental Health of transgender and gender diverse youth. Ann Rev Clin Psychol. 2023;19:207–32. 36608332 10.1146/annurev-clinpsy-072220-020326 PMC9936952

[26] de Graaf NM, Huisman B, Cohen-Kettenis PT, Twist J, Hage K, Carmichael P, et al. Psychological functioning in non-binary identifying adolescents and adults. J Sex Marital Ther. 2021;47:773–84. 34344272 10.1080/0092623X.2021.1950087

[27] Chew D, Tollit MA, Poulakis Z, Zwickl S, Cheung AS. Youths with a non-binary gender identity: a review of their sociodemographic and clinical profile. Rev Lancet Child Adolesc Health. 2020;4:322–52. 10.1016/S2352-4642(19)30403-1 31978373

[28] Page MJ, McKenzie JE, Bossuyt PM, Boutron I, Hoffmann TC, Mulrow CD et al. The PRISMA 2020 statement: an updated guideline for reporting systematic reviews. BMJ. 2021;372. 10.1136/bmj.n71 PMC8005924 33782057

[29] Moola S, Munn Z, Tufanaru C, Aromataris E, Sears K, Sfetcu R, et al. Chapter 7: systematic reviews of etiology and risk. In: Aromataris E, Munn Z, editors. JBI Manual for evidence synthesis. JBI; 2020.

[30] Goplen CM, Verbeek W, Kang SH, Jones CA, Voaklander DC, Churchill TA et al. Preoperative opioid use is associated with worse patient outcomes after total joint arthroplasty: a systematic review and meta-analysis. BMC Musculoskelet Disord. 2019;20. 10.1186/s12891-019-2619-8 PMC6525974 31103029

[31] Melo G, Dutra KL, Rodrigues Filho R, Ortega AOL, Porporatti AL, Dick B, et al. Association between psychotropic medications and presence of sleep bruxism: a systematic review. J Oral Rehabil. 2018;45:545–54. 29663484 10.1111/joor.12633

[32] Borenstein M, Hedges LV, Higgins JPT, Rothstein HR. Comprehensive Meta-Analysis Version 4. 2022.

[33] Cohen J. Statistical Power Analysis for the behavioral sciences. 2nd ed. Lawrence Erlbaum Associates; 1988.

[34] Higgins JPT, Thompson SG, Deeks JJ, Altman DG. Measuring inconsistency in meta-analyses. BMJ. 2003;327:557–60. 12958120 10.1136/bmj.327.7414.557 PMC192859

[35] Olsavsky AL, Grannis C, Bricker J, Chelvakumar G, Indyk JA, Leibowitz SF, et al. Associations among gender-affirming hormonal interventions, Social Support, and Transgender adolescents’ Mental Health. J Adolesc Health. 2023;72:860–8. 37029048 10.1016/j.jadohealth.2023.01.031

[36] Thorne N, Witcomb GL, Nieder T, Nixon E, Yip A, Arcelus J. A comparison of mental health symptomatology and levels of social support in young treatment seeking transgender individuals who identify as binary and non-binary. 2018. 10.1080/15532739.2018.1452660 10.1080/15532739.2018.1452660 PMC6830974 32999610

[37] Ciria-Barreiro E, Moreno-Maldonado C, Rivera F, Moreno C. A comparative study of Health and Wellbeing among Cisgender and Binary and Nonbinary Transgender adolescents in Spain. LGBT Health. 2021;8:536–44. 34648726 10.1089/lgbt.2020.0477

[38] Childs AW, Kaufman CC, Olezeski CL. How is everyone doing? Baseline psychological distress and adaptive functioning among Transgender, Nonbinary, and Cis youth presenting for intensive outpatient Psychiatric Services. Psychol Serv. 2022;19:541–50. 34292007 10.1037/ser0000573

[39] Rusow JA, Hidalgo MA, Calvetti S, Quint M, Wu S, Bray BC, et al. Health and service utilization among a sample of gender-diverse youth of color: the TRUTH study. BMC Public Health. 2022;22:1–13. 36496355 10.1186/s12889-022-14585-9 PMC9737736

[40] Goldberg DP, Williams P. A user’s guide to the General Health Questionnaire. NFER-Nelson; 1988.

[41] Reynolds CR, Kamphaus RW. Behavior assessment system for children, third edition (BASC-3) manual. Bloomington: Pearson; 2015.

[42] Haugland S, Wold B. Subjective health complaints in adolescence—reliability and validity of survey methods. J Adolesc. 2001;24:611–24. 11676508 10.1006/jado.2000.0393

[43] Asner-Self KK, Schreiber JB, Marotta SA. A cross-cultural analysis of the brief symptom inventory-18. Cultur Divers Ethnic Minor Psychol. 2006;12:367–75. 16719583 10.1037/1099-9809.12.2.367

[44] McKay TR, Watson RJ. Gender expansive youth disclosure and mental health: clinical implications of gender identity disclosure. Psychol Sex Orientat Gend Divers. 2020;7:66–75. 33855103 10.1037/sgd0000354 PMC8043602

[45] Garthe RC, Blackburn AM, Kaur A, Sarol JN, Goffnett J, Rieger A, et al. Suicidal ideation among transgender and gender expansive youth: mechanisms of risk. Transgend Health. 2022;7:416–22. 36644491 10.1089/trgh.2021.0055 PMC9829150

[46] Jardas E, Ladd BA, Maheux AJ, Choukas-Bradley S, Salk RH, Thoma BC. Testing the minority stress model across gender identity, race, and ethnicity among U.S. gender minority adolescents. J Psychopathol Clin Sci. 2023;132:542–54. 37261780 10.1037/abn0000834 PMC10659140

[47] Parodi KB, Holt MK, Green JG, Katz-Wise SL, Shah TN, Kraus AD, et al. Associations between school-related factors and mental health among transgender and gender diverse youth. J Sch Psychol. 2022;90:135–49. 34969484 10.1016/j.jsp.2021.11.004

[48] Peng K, Zhu X, Gillespie A, Wang Y, Gao Y, Xin Y, et al. Self-reported rates of abuse, neglect, and bullying experienced by transgender and gender-nonbinary adolescents in China. JAMA Netw Open. 2019;2:1–12. 10.1001/jamanetworkopen.2019.11058 PMC6735403 31490542

[49] Price-Feeney M, Green AE, Dorison S. Understanding the Mental Health of Transgender and Nonbinary Youth. J Adolesc Health. 2020;66:684–90. 31992489 10.1016/j.jadohealth.2019.11.314

[50] Srivastava A, Rusow JA, Goldbach JT. Differential risks for suicidality and mental health symptoms among transgender, nonbinary, and cisgender sexual minority youth accessing crisis services. Transgend Health. 2021;6. 10.1089/trgh.2020.0034 PMC7906236 33644322

[51] Sterzing PR, Ratliff GA, Gartner RE, McGeough BL, Johnson KC. Social Ecological Correlates of Polyvictimization among a National Sample of Transgender, Genderqueer, and cisgender sexual minority adolescents. Child Abuse Negl. 2017;67:1–12. 28226283 10.1016/j.chiabu.2017.02.017

[52] Wang Y, Yu H, Yang Y, Drescher J, Li R, Yin W, et al. Mental Health Status of Cisgender and gender-diverse secondary school students in China. JAMA Netw Open. 2020;3:e2022796. 33107922 10.1001/jamanetworkopen.2020.22796 PMC7592029

[53] Kaltiala R, Heino E, Marttunen M, Fröjd S. Family characteristics, Transgender Identity and emotional symptoms in adolescence: a population survey study. Int J Environ Res Public Health. 2023;20. 10.3390/ijerph20042948 PMC9963798 36833645

[54] Radloff LS, The CES-D, Scale. A self-report depression scale for research in the general population. Appl Psychol Meas. 1977;1:385–401.

[55] Kroenke K, Spitzer RL, Williams JBW. The patient health questionnaire-2: validity of a two-item depression screener. Med Care. 2003;41:1284–92. 14583691 10.1097/01.MLR.0000093487.78664.3C

[56] Löwe B, Kroenke K, Herzog W, Gräfe K. Measuring depression outcome with a brief self-report instrument: sensitivity to change of the patient health questionnaire (PHQ-9). J Affect Disord. 2004;81:61–6. 15183601 10.1016/S0165-0327(03)00198-8

[57] Kovacs M. The children’s depression, inventory (CDI). Psychopharmacol Bull. 1985;21:995–8. 4089116

[58] Zigmond AS, Snaith RP. The hospital anxiety and depression scale. Acta Psychiatr Scand. 1983;67:361–70. 6880820 10.1111/j.1600-0447.1983.tb09716.x

[59] Kann L, McManus T, Harris WA, Shanklin SL, Flint KH, Queen B, et al. Youth risk behavior surveillance — United States, 2017. MMWR Surveill Summ. 2018;67:1. 29902162 10.15585/mmwr.ss6708a1 PMC6002027

[60] Spitzer RL, Kroenke K, Williams JBW, Löwe B. A brief measure for assessing generalized anxiety disorder. Arch Intern Med. 2006;166:1092. 16717171 10.1001/archinte.166.10.1092

[61] Birmaher B, Brent DA, Chiappetta L, Bridge J, Monga S, Baugher M. Psychometric properties of the screen for child anxiety related emotional disorders (SCARED): a replication study. J Am Acad Child Adolesc Psychiatry. 1999;38:1230–6. 10517055 10.1097/00004583-199910000-00011

[62] Thoma BC, Salk RH, Choukas-Bradley S, Goldstein TR, Levine MD, Marshal MP. Suicidality disparities between Transgender and Cisgender adolescents. Pediatrics. 2019;144. 10.1542/peds.2019-1183 PMC7011156 31611339

[63] Thoma BC, Rezeppa TL, Choukas-Bradley S, Salk RH, Marshal MP. Disparities in childhood abuse between transgender and cisgender adolescents. Pediatrics. 2021;148. 10.1542/peds.2020-016907 PMC8344346 34226247

[64] Toomey RB, Syvertsen AK, Shramko M. Transgender Adolesc Suicide Behav. 2018;142:20174218. 10.1542/peds.2017-4218 PMC6317573 30206149

[65] Meyer IH, Blosnich JR, Choi SK, Harper GW, Russell ST. Suicidal behavior and coming out milestones in three cohorts of sexual minority adults. LGBT Health. 2021;8:340–8. 34096796 10.1089/lgbt.2020.0466 PMC8252903

[66] Borenstein M, Hedges LV, Higgins JPT, Rothstein HR. Introduction to meta-analysis. 2nd edition. Wiley; 2021.

[67] Holt V, Skagerberg E, Dunsford M. Young people with features of gender dysphoria: demographics and associated difficulties. Clin Child Psychol Psychiatry. 2016;21:108–18. 25431051 10.1177/1359104514558431

[68] Spack NP, Edwards-Leeper L, Feldman HA, Leibowitz S, Mandel F, Diamond DA, et al. Children and adolescents with gender identity disorder referred to a pediatric medical center. Pediatrics. 2012;129:418–25. 22351896 10.1542/peds.2011-0907

[69] Khatchadourian K, Amed S, Metzger DL. Clinical management of youth with gender dysphoria in Vancouver. J Pediatr. 2014;164:906–11. 24315505 10.1016/j.jpeds.2013.10.068

[70] Olson J, Schrager SM, Belzer M, Simons LK, Clark LF. Baseline physiologic and psychosocial characteristics of transgender youth seeking care for gender dysphoria. J Adolesc Health. 2015;57:374–80. 26208863 10.1016/j.jadohealth.2015.04.027 PMC5033041

[71] Pellicane MJ, Ciesla JA. Associations between minority stress, depression, and suicidal ideation and attempts in transgender and gender diverse (TGD) individuals: systematic review and meta-analysis. Clin Psychol Rev. 2022;91. 10.1016/j.cpr.2021.102113 34973649

[72] Mezza F, Mezzalira S, Pizzo R, Maldonato NM, Bochicchio V, Scandurra C. Minority stress and mental health in European transgender and gender diverse people: a systematic review of quantitative studies. Clin Psychol Rev. 2024;107:102358. 37995435 10.1016/j.cpr.2023.102358

[73] Mason A, Crowe E, Haragan B, Smith S, Kyriakou A. Gender dysphoria in young people: a model of chronic stress. Horm Res Paediatr. 2021;94:340–51. 10.1159/000520361 34673639

[74] DuBois LZ, Puckett JA, Jolly D, Powers S, Walker T, Hope DA et al. Gender minority stress and diurnal cortisol profiles among transgender and gender diverse people in the United States. Horm Behav. 2024;159. 10.1016/j.yhbeh.2023.105473 38190769

[75] Johnson KC, LeBlanc AJ, Deardorff J, Bockting WO. Invalidation experiences among non-binary adolescents. J Sex Res. 2020;57:222–33. 31070487 10.1080/00224499.2019.1608422

[76] Paz Galupo M, Pulice-Farrow L, Pehl E. There is nothing to do about it: nonbinary individuals’ experience of gender dysphoria. Transgend Health. 2021;6:101–10. 34414266 10.1089/trgh.2020.0041 PMC8363999

[77] Murawsky S. The struggle with transnormativity: non-binary identity work, embodiment desires, and experience with gender dysphoria. Soc Sci Med. 2023;327:115953. 37156019 10.1016/j.socscimed.2023.115953

[78] Taliaferro LA, McMorris BJ, Rider GN, Eisenberg ME. Risk and protective factors for self-harm in a Population-based sample of Transgender Youth. Archives Suicide Res. 2019;23:203–21. 10.1080/13811118.2018.1430639 PMC6102088 29461934

[79] Liu RT, Sheehan AE, Walsh RFL, Sanzari CM, Cheek SM, Hernandez EM. Prevalence and correlates of non-suicidal self-injury among lesbian, gay, bisexual, and transgender individuals: a systematic review and meta-analysis. Clin Psychol Rev. 2019;74:101783. 31734440 10.1016/j.cpr.2019.101783 PMC6896220

[80] Rhoades H, Rusow JA, Bond D, Lanteigne A, Fulginiti A, Goldbach JT. Homelessness, mental health and suicidality among LGBTQ youth accessing crisis services. Child Psychiatry Hum Dev. 2018;49:643–51. 29322361 10.1007/s10578-018-0780-1

[81] Noralla N. Access denied: a qualitative Study on transgender health policy in Egypt. Soc Sci Med. 2024;348. 10.1016/j.socscimed.2024.116867 38581813

[82] Conlin SE, Douglass RP, Larson-Konar DM, Gluck MS, Fiume C, Heesacker M. Exploring Nonbinary gender identities: a qualitative content analysis. J LGBT Issues Couns. 2019;13:114–33.

[83] Ryan C, Russell ST, Huebner D, Diaz R, Sanchez J. Family acceptance in adolescence and the health of LGBT young adults. J Child Adolesc Psychiatric Nurs. 2010;23:205–13. 10.1111/j.1744-6171.2010.00246.x 21073595

