# Supplementary material for: Mental health of non-binary youth: a systematic review and meta-analysis
Source: Child Adolesc Psychiatry Ment Health. 2024 Oct 9;18:126. doi: 10.1186/s13034-024-00822-z (PMC11465615; doi:10.1186/s13034-024-00822-z)
Supplement: Supplementary file 1 — Supplementary Material 1 [file 13034_2024_822_MOESM1_ESM.docx]

**Additional file 1**

**Supplementary Information**

**Mental health of non-binary youth: a systematic review and meta-analysis**

Diana Klinger, Sofia-Marie Oehlke, Stefan Riedl, Ken Eschbaum, Heidi Elisabeth Zesch, Andreas Karwautz, Paul L. Plener, Oswald D. Kothgassner

**Table S1**

*Prisma 2020 Checklist*

| **Section and Topic** | **Item #** | **Checklist item** | **Location where item is reported** |
| --- | --- | --- | --- |
| **TITLE** | | |  |
| Title | 1 | Identify the report as a systematic review. | Title |
| **ABSTRACT** | | |  |
| Abstract | 2 | See the PRISMA 2020 for Abstracts checklist. | N/A |
| **INTRODUCTION** | | |  |
| Rationale | 3 | Describe the rationale for the review in the context of existing knowledge. | Background |
| Objectives | 4 | Provide an explicit statement of the objective(s) or question(s) the review addresses. | Background |
| **METHODS** | | |  |
| Eligibility criteria | 5 | Specify the inclusion and exclusion criteria for the review and how studies were grouped for the syntheses. | Methods/ Inclusion and exclusion criteria |
| Information sources | 6 | Specify all databases, registers, websites, organisations, reference lists and other sources searched or consulted to identify studies. Specify the date when each source was last searched or consulted. | Methods/ Data sources and search strategy |
| Search strategy | 7 | Present the full search strategies for all databases, registers and websites, including any filters and limits used. | Methods/ Data sources and search strategy |
| Selection process | 8 | Specify the methods used to decide whether a study met the inclusion criteria of the review, including how many reviewers screened each record and each report retrieved, whether they worked independently, and if applicable, details of automation tools used in the process. | Methods/Screening and selection process |
| Data collection process | 9 | Specify the methods used to collect data from reports, including how many reviewers collected data from each report, whether they worked independently, any processes for obtaining or confirming data from study investigators, and if applicable, details of automation tools used in the process. | Methods/Data extraction |
| Data items | 10a | List and define all outcomes for which data were sought. Specify whether all results that were compatible with each outcome domain in each study were sought (e.g. for all measures, time points, analyses), and if not, the methods used to decide which results to collect. | Methods/Data extraction |
|  | 10b | List and define all other variables for which data were sought (e.g. participant and intervention characteristics, funding sources). Describe any assumptions made about any missing or unclear information. | Methods/Data extraction |
| Study risk of bias assessment | 11 | Specify the methods used to assess risk of bias in the included studies, including details of the tool(s) used, how many reviewers assessed each study and whether they worked independently, and if applicable, details of automation tools used in the process. | Methods/Quality assessment |
| Effect measures | 12 | Specify for each outcome the effect measure(s) (e.g. risk ratio, mean difference) used in the synthesis or presentation of results. | Methods/ Data synthesis and statistical analysis |
| Synthesis methods | 13a | Describe the processes used to decide which studies were eligible for each synthesis (e.g. tabulating the study intervention characteristics and comparing against the planned groups for each synthesis (item #5)). | Methods/ Data synthesis and statistical analysis |
|  | 13b | Describe any methods required to prepare the data for presentation or synthesis, such as handling of missing summary statistics, or data conversions. | Methods/ Data synthesis and statistical analysis |
|  | 13c | Describe any methods used to tabulate or visually display results of individual studies and syntheses. | Methods/ Data synthesis and statistical analysis |
|  | 13d | Describe any methods used to synthesize results and provide a rationale for the choice(s). If meta-analysis was performed, describe the model(s), method(s) to identify the presence and extent of statistical heterogeneity, and software package(s) used. | Methods/ Data synthesis and statistical analysis |
|  | 13e | Describe any methods used to explore possible causes of heterogeneity among study results (e.g. subgroup analysis, meta-regression). | Methods/ Data synthesis and statistical analysis |
|  | 13f | Describe any sensitivity analyses conducted to assess robustness of the synthesized results. | Methods/ Data synthesis and statistical analysis |
| Reporting bias assessment | 14 | Describe any methods used to assess risk of bias due to missing results in a synthesis (arising from reporting biases). | N/A |
| Certainty assessment | 15 | Describe any methods used to assess certainty (or confidence) in the body of evidence for an outcome. | N/A |
| **RESULTS** | | |  |
| Study selection | 16a | Describe the results of the search and selection process, from the number of records identified in the search to the number of studies included in the review, ideally using a flow diagram. | Results/Study selection, Figure 1 |
|  | 16b | Cite studies that might appear to meet the inclusion criteria, but which were excluded, and explain why they were excluded. | Results/Study selection, Table S3 |
| Study characteristics | 17 | Cite each included study and present its characteristics. | Results/Study characteristics, Table 1 |
| Risk of bias in studies | 18 | Present assessments of risk of bias for each included study. | Results/Quality assessment results, Table S2 |
| Results of individual studies | 19 | For all outcomes, present, for each study: (a) summary statistics for each group (where appropriate) and (b) an effect estimate and its precision (e.g. confidence/credible interval), ideally using structured tables or plots. | Results |
| Results of syntheses | 20a | For each synthesis, briefly summarise the characteristics and risk of bias among contributing studies. | Results |
|  | 20b | Present results of all statistical syntheses conducted. If meta-analysis was done, present for each the summary estimate and its precision (e.g. confidence/credible interval) and measures of statistical heterogeneity. If comparing groups, describe the direction of the effect. | Results, Figures 2-4 |
|  | 20c | Present results of all investigations of possible causes of heterogeneity among study results. | Results |
|  | 20d | Present results of all sensitivity analyses conducted to assess the robustness of the synthesized results. | Results/Sensitivity analysis |
| Reporting biases | 21 | Present assessments of risk of bias due to missing results (arising from reporting biases) for each synthesis assessed. | N/A |
| Certainty of evidence | 22 | Present assessments of certainty (or confidence) in the body of evidence for each outcome assessed. | N/A |
| **DISCUSSION** | | |  |
| Discussion | 23a | Provide a general interpretation of the results in the context of other evidence. | Discussion |
|  | 23b | Discuss any limitations of the evidence included in the review. | Discussion/Strengths and limitations |
|  | 23c | Discuss any limitations of the review processes used. | Discussion/Strengths and limitations |
|  | 23d | Discuss implications of the results for practice, policy, and future research. | Discussion |
| **OTHER INFORMATION** | | |  |
| Registration and protocol | 24a | Provide registration information for the review, including register name and registration number, or state that the review was not registered. | N/A |
|  | 24b | Indicate where the review protocol can be accessed, or state that a protocol was not prepared. | N/A |
|  | 24c | Describe and explain any amendments to information provided at registration or in the protocol. | N/A |
| Support | 25 | Describe sources of financial or non-financial support for the review, and the role of the funders or sponsors in the review. | N/A |
| Competing interests | 26 | Declare any competing interests of review authors. | N/A |
| Availability of data, code and other materials | 27 | Report which of the following are publicly available and where they can be found: template data collection forms; data extracted from included studies; data used for all analyses; analytic code; any other materials used in the review. | N/A |

**Table S2**

*Study quality assessment*

**Legend**

Q1. Were the criteria for inclusion in the sample clearly defined?

Q2. Were the study subjects and the setting described in detail?

Q3. Was the exposure measured in a valid and reliable way?

Q4. Were objective, standard criteria used for measurement of the condition?

Q5. Were confounding factors identified?

Q6. Were strategies to deal with confounding factors stated?

Q7. Were the outcomes measured in a valid and reliable way?

Q8. Was appropriate statistical analysis used?

Y = yes, N = no; U = unclear; N/A = not applicable

| **Study Name** | **Q1** | **Q2** | **Q3** | **Q4** | **Q5** | **Q6** | **Q7** | **Q8** | **Total Score** | **%** |
| --- | --- | --- | --- | --- | --- | --- | --- | --- | --- | --- |
| Aparicio-Garcia et al. (2018) | N | Y | N/A | Y | Y | Y | Y | Y | **6** | **75%** |
| Childs et al. (2022) | Y | Y | N/A | Y | N | N | Y | Y | **5** | **63%** |
| Ciria-Barreiro et al. (2021) | Y | Y | N/A | Y | N | N | Y | Y | **5** | **63%** |
| Clark et al. (2018) | Y | Y | N/A | Y | Y | Y | U | Y | **6** | **75%** |
| Garthe et al. (2022) | Y | Y | N/A | N | Y | Y | N | Y | **5** | **63%** |
| Jardas et al. (2023) | Y | Y | N/A | Y | Y | Y | Y | Y | **7** | **88%** |
| Kaltiala et al. (2023) | Y | Y | N/A | N | Y | Y | Y | Y | **6** | **75%** |
| McKay & Watson (2020) | Y | Y | N/A | Y | Y | Y | Y | Y | **7** | **88%** |
| Meyer et al. (2021) | Y | Y | N/A | Y | Y | Y | Y | Y | **7** | **88%** |
| Olsavsky et al. (2023) | Y | Y | Y | Y | Y | Y | Y | Y | **8** | **100%** |
| Parodi et al. (2022) | Y | Y | N/A | Y | Y | Y | Y | Y | **7** | **88%** |
| Peng et al. (2019) | Y | Y | N/A | Y | Y | Y | Y | Y | **7** | **88%** |
| Price-Feeney et al. (2020) | Y | Y | N/A | Y | Y | Y | N | Y | **6** | **75%** |
| Rimes et al. (2017) | N | Y | N/A | Y | N | N | Y | Y | **4** | **50%** |
| Rusow et al. (2022) | Y | Y | N/A | Y | N | N | Y | Y | **5** | **63%** |
| Srivastava et al. (2021) | Y | Y | N/A | Y | Y | Y | Y | Y | **7** | **88%** |
| Sterzing et al. (2017) | Y | Y | N/A | N | N | N | Y | Y | **4** | **50%** |
| Thoma et al. (2019) | Y | Y | N/A | Y | Y | Y | Y | Y | **7** | **88%** |
| Thorne et al. (2018) | Y | Y | N/A | Y | N | N | Y | Y | **5** | **63%** |
| Toomey et al. (2018) | N | Y | N/A | Y | Y | Y | N | Y | **5** | **63%** |
| Wang et al. (2020) | Y | Y | N/A | Y | Y | Y | Y | Y | **7** | **88%** |
| **%** | **86%** | **100%** | **5%** | **86%** | **71%** | **71%** | **81%** | **100%** |  |  |

**Table S3**

*Reasons for exclusion of studies from the systematic literature search, after screening for title and abstract*

| **Study** | **Reason for exclusion** |
| --- | --- |
| Almazan & Keuroghlian (2021) | Inappropriate study population (includes individuals >25 years of age) |
| Anderssen et al. (2020) | Inappropriate study population (includes individuals >25 years of age) |
| Attebery-Ash et al. (2021) | Inappropriate study groups (“Trans other” includes anyone, who did not identify as “Transgender man”, or “Transgender woman”) |
| Barr et al. (2016) | Inappropriate study population (includes individuals >25 years of age) |
| Beckwith et al. (2019) | Inappropriate study population (includes individuals >25 years of age) |
| Berry et al. (2023) | Inappropriate study population (includes individuals >25 years of age) |
| Blackshaw et al. (2023) | Inappropriate study groups (comparison group may include both cisgender and transgender individuals) |
| Bowling et al. (2020) | Inappropriate study population (includes individuals >25 years of age) |
| Bretherton et al. (2021) | Inappropriate study population (includes individuals >25 years of age) |
| Butler et al. (2019) | Inappropriate study groups (“Other” includes anyone, who did not identify as “Trans”, “Male”, or “Female”) |
| Buttazoni et al. (2021) | Inappropriate study groups (non-binary group includes transgender individuals) |
| Chen et al. (2023) | Inappropriate study population (includes individuals >25 years of age) |
| Coburn et al. (2022) | Inappropriate study population (includes individuals >25 years of age) |
| del Río-González et al. (2021) | Inappropriate study population (includes individuals >25 years of age) |
| Ding et al. (2023) | Inappropriate study groups (comparison group may include both cisgender and transgender individuals) |
| Dion et al. (2022) | Inappropriate study groups (comparison group includes both cisgender and transgender individuals) |
| Durbeej et al. (2021) | Inappropriate study groups (comparison group includes both cisgender and transgender individuals) |
| Ferlatte et al. (2020) | Insufficient data to calculate effect sizes |
| Fontanari et al. (2020) | Insufficient data to calculate effect sizes |
| Fredriksen Goldsen et al. (2022) | Inappropriate study population (includes individuals >25 years of age) |
| Gower et al. (2022) | Inappropriate study groups (comparison group includes both cisgender and transgender individuals) |
| Green et al. (2022) | Insufficient data to calculate effect sizes |
| Haider et al. (2023) | Inappropriate study groups (comparison group includes both cisgender and transgender individuals) |
| Jadva et al. (2022) | Inappropriate study groups (comparison group includes both cisgender and transgender individuals) |
| Jarett et al. (2021) | Inappropriate study population (includes individuals >25 years of age) |
| Kachen et al. (2021) | Inappropriate study population (includes individuals >25 years of age) |
| Kattari et al. (2020) | Inappropriate study population (includes individuals >25 years of age) |
| Kingsbury & Arim (2023) | Inappropriate study groups (comparison group includes both non-binary and transgender individuals) |
| Lee et al. (2023) | Inappropriate study groups (comparison group may include both cisgender and transgender individuals) |
| Levant et al. (2019) | Inappropriate study population (includes individuals >25 years of age) |
| Newcomb et al. (2020) | Inappropriate study population (includes individuals >25 years of age) |
| Rutherford et al. (2021) | Inappropriate study population (includes individuals >25 years of age) |
| Rutter et al. (2021) | Insufficient data to calculate effect sizes |
| Scandurra et al. (2021) | Inappropriate study population (separate analysis regarding non-binary people includes individuals >25 years of age) |
| Small et al. (2022) | Inappropriate study population (includes individuals >25 years of age) |
| Smout et al. (2023) | Inappropriate study groups (comparison group includes both cisgender and transgender individuals) |
| Solanki et al. (2022) | Inappropriate study population (includes individuals >25 years of age) |
| Sun et al. (2023) | Inappropriate study groups (comparison group is transgender females) |
| Tan et al. (2020) | Inappropriate study population (includes individuals >25 years of age) |
| Tordoff et al. (2022) | Inappropriate study groups (comparison group are cisgender or transgender males) |
| Turner et al. (2022) | Inappropriate study groups (non-binary group includes transgender individuals) |
| Vázquez et al. (2023) | Inappropriate study population (includes individuals >25 years of age) |
| Veale et al. (2017) | Reported data from already included study |
| Veale et al. (2022) | Inappropriate study population (includes individuals >25 years of age) |
| Wathelet et al. (2020) | Inappropriate study groups (“Other” includes anyone, who did not identify “Male”, or “Female”) |
| Wathelet et al. (2022) | Inappropriate study groups (“Other” includes anyone, who did not identify “Male”, or “Female”) |
| Williams et al. (2023) | Insufficient data to calculate effect sizes |
| Yee et al. (2022) | Inappropriate study population (includes individuals >25 years of age) |
